# Outbreaks of fluconazole-resistant *Candida parapsilosis* are driven by low-biofilm-producing isolates that emerge under host selection

**DOI:** 10.1371/journal.pbio.3003973

**Published:** 2026-09-01

**Authors:** Farnaz Daneshnia, Deepika Gunasekaran, Sean Bergin, Lisa Lombardi, Austin M. Perry, Liuyang Cai, Louise A. Walker, Tibor Nemeth, Süleyha Hilmioglu-Polat, Letal I. Salzberg, Arefeh Ebadati, Tobias Köhler, Gabriel Braune, João N. de Almeida, Giuseppina Caggiano, Julianne V. Kus, Pegah Mosharaf Ghahfarokhy, Julieta Munoz, Daniel J. Floyd, Diego Fuentes-Palacios, Samuel M. Gonçalves, Relber A. Gonçales, Mostafa Salehi, Jigar V. Desai, Agostinho Carvalho, Shenglin Mei, Carol A. Munro, Alex Hopke, Toni Gabaldón, Attila Gacser, Oliver Kurzai, Geraldine Butler, David S. Perlin, Wenjie Fang, Clarissa J. Nobile, Michael K. Mansour, Amir Arastehfar

**Affiliations:** 1 Institute of Biodiversity and Ecosystem Dynamics (IBED), University of Amsterdam, Amsterdam, The Netherlands; 2 Division of Infectious Diseases, Massachusetts General Hospital, Boston, Massachusetts, United States of America; 3 Department of Molecular and Cell Biology, School of Natural Sciences, University of California Merced, Merced, California, United States of America; 4 School of Biomolecular and Biomedical Science, Conway Institute, University College Dublin, Dublin, Ireland; 5 Department of Biology, University of Pisa, Pisa, Italy; 6 Quantitative and Systems Biology Graduate Program, University of California Merced, Merced, California, United States of America; 7 Department of Dermatology, Shanghai Key Laboratory of Molecular Medical Mycology, Shanghai Changzheng Hospital, Naval Medical University, Shanghai, China; 8 The Center for Fungal Infectious Diseases Basic Research and Innovation of Medicine and Pharmacy, Ministry of Education, Shanghai, China; 9 Institute of Medical Sciences, University of Aberdeen, Aberdeen, United Kingdom; 10 Department of Biotechnology and Microbiology, Faculty of Science and Informatics, University of Szeged, Szeged, Hungary; 11 Department of Medical Microbiology, Ege University Faculty of Medicine, Izmir, Türkiye; 12 Health Sciences Research Institute, University of California Merced, Merced, California, United States of America; 13 Institute for Hygiene and Microbiology, University of Würzburg, Würzburg, Germany; 14 Special Mycology Laboratory, Federal University of São Paulo, São Paulo, Brazil; 15 Clinical Laboratory, Hospital Israelita Albert Einstein, São Paulo, Brazil; 16 Interdisciplinary Department of Medicine, Hygiene Section, University of Bari Aldo Moro, Bari, Italy; 17 Public Health Ontario, Toronto, Ontario, Canada; 18 Department of Laboratory Medicine and Pathobiology, University of Toronto, Toronto, Ontario, Canada; 19 Life Sciences Programme, Supercomputing Center (BSC-CNS), Barcelona, Spain; 20 Institute for Research in Biomedicine (IRB Barcelona), The Barcelona Institute of Science and Technology, Barcelona, Spain; 21 Life and Health Sciences Research Institute (ICVS), School of Medicine, University of Minho, Braga, Portugal; 22 ICVS/3B’s-PT Government Associate Laboratory, Guimarães/Braga, Portugal; 23 Department of Industrial Engineering, Faculty of K.N., Toosi University of Technology, Tehran, Iran; 24 Center for Discovery and Innovation, Hackensack Meridian Health, Nutley, New Jersey, United States of America; 25 Fralin Biomedical Research Institute, Virginia Tech FBRI Cancer Research Center, Washington, District of Columbia, United States of America; 26 Department of Biomedical Sciences and Pathobiology, College of Veterinary Medicine, Virginia Tech, Blacksburg, Virginia, United States of America; 27 Department of Biomedical Sciences, East Tennessee State University Quillen College of Medicine, Center of Excellence in Inflammation, Infectious Disease and Immunity, East Tennessee State University, Johnson City, Tennessee, United States of America; 28 Catalan Institution for Research and Advanced Studies, Barcelona, Spain; 29 Centro de Investigación Biomédica en Red de Enfermedades Infecciosas (CIBERINFEC), Barcelona, Spain; 30 National Reference Center for Invasive Fungal Infections NRZMyk, Leibniz Institute for Natural Product Research and Infection Biology—Hans-Knoell-Institute, Jena, Germany; 31 Georgetown University Lombardi Comprehensive Cancer Center, Washington, United States of America; 32 Center for Cellular and Biomolecular Machines, University of California Merced, Merced, California, United States of America; 33 Department of Medicine, Harvard Medical School, Boston, Massachusetts, United States of America; 34 University Hospital Wuerzburg, Medical Hospital II, Würzburg, Germany; University of Utah, UNITED STATES OF AMERICA

## Abstract

*Candida parapsilosis* is a major human fungal pathogen, with recent global outbreaks driven by fluconazole-resistant (FLCR-Cp) isolates that are difficult to eradicate and associated with poor clinical outcomes. However, the microbial traits enabling persistence of these outbreak lineages remain poorly defined. Here, we show that FLCR-Cp isolates responsible for prolonged, multi-country outbreaks consistently exhibit a striking low-biofilm-producing (LBP) phenotype. Contrary to the prevailing view that robust biofilm formation promotes persistence, LBP strains displayed enhanced stress tolerance, increased cell wall masking, and reduced immune recognition. These traits conferred resistance to neutrophil and macrophage killing and enhanced survival in immune cell-rich organs during systemic infection. Genome-wide transcriptomic profiling revealed extensive metabolic and regulatory rewiring in LBP strains. Whole-genome sequencing (WGS) of a global isolate collection further demonstrated that the LBP phenotype has emerged independently multiple times, supporting convergent evolution under host selection. Functional genomic analyses suggest that biofilm attenuation arises through multigenic changes, and disruption of key biofilm-associated transcriptional regulators enhanced fitness during immune interactions. Together, our findings overturn the assumption that robust biofilm formation drives outbreak persistence and instead identify biofilm attenuation as an adaptive tradeoff that promotes immune evasion and long-term survival. These results redefine our understanding of *C. parapsilosis* adaptation during healthcare-associated outbreaks and shift attention toward host-driven evolutionary processes than environmental persistence alone.

## Abstract 2

Drug-resistant Candida parapsilosis is an increasing problem in hospitals, but what traits help fungus survive? This study shows that reduced biofilm formation is repeatedly selected for during infection and helps C. parapsilosis evade immune defenses and persist in the host.

## Introduction

Invasive fungal infections impose a major global health and economic burden, costing billions of U.S. dollars annually [1] and causing more than 3.5 million deaths each year, with nearly 1 million deaths attributed to yeast species of the *Candida* genus [2]. *Candida parapsilosis* ranks among the top three causes of candidemia worldwide, with prevalence varying by patient population and geographic region [3,4]. The *C. parapsilosis* species complex comprises three genetically distinct but phenotypically similar species: *C. parapsilosis* sensu *stricto*, *Candida orthopsilosis*, and *Candida metapsilosis* [5]. Among these, *C. parapsilosis* sensu *stricto* is the most virulent and is primarily responsible for healthcare-associated outbreaks and device colonization [6].

Recent epidemiological reports from Europe, North America, the Mediterranean, and Southeast Asia have documented an alarming rise in fluconazole-resistant (FLCR) *C. parapsilosis* isolates, driving persistent hospital outbreaks [3,4,7,8]. Infections caused by FLCR isolates exhibit more than twice the mortality of infections with susceptible strains [9,10], and multidrug-resistant (MDR) lineages have emerged from these FLCR backgrounds [11]. Notably, FLCR isolates frequently infect antifungal-naïve patients [4] and persist in healthcare environments despite rigorous infection control measures [10]. Molecular epidemiology studies have demonstrated that isolates recovered from patient blood, hospital surfaces, and healthcare workers’ hands are often genetically indistinguishable [10], implicating both environmental and host-associated reservoirs in sustaining transmission. Yet, the microbial traits that enable these resistant lineages to persist remain poorly defined.

Biofilm formation—the capacity of microbial cells to assemble structured, extracellular matrix-encased communities—has long been considered a central persistence factor in pathogenic *Candida* species, including *Candida albicans* and *C. parapsilosis* [3,4,12]. Fungal biofilms contribute to an estimated $43 billion in annual healthcare costs [13] due to their tolerance to antifungals, disinfectants, and immune defenses. Biofilm-derived cells can exhibit enhanced virulence [14] and facilitate colonization of new niches [12,13]. Consistent with observations in bacterial pathogens, biofilm formation in *Candida* species is positively associated with clinical persistence, therapeutic failure, and mortality [15,16]. Consequently, robust biofilm production has been widely assumed to underlie the success of outbreak lineages.

Under this prevailing model, the persistence of FLCR *C. parapsilosis*—where identical genotypes circulate between patients and hospital environments [4,17]—is typically attributed to biofilm-mediated resilience. Therefore, repeated exposure to host tissues, immune pressures, and environmental stressors would be expected to select for increasingly robust biofilm formation. Indeed, both clinical [18] and experimental evolution studies [19] have demonstrated that fungal pathogens can rapidly adapt virulence-associated traits to enhance fitness within complex host niches. However, the microbiological drivers and genetic mechanisms sustaining persistent *C. parapsilosis* outbreaks remain unresolved.

Here, we integrate phenotypic, transcriptomic, and genomic analyses to uncover an unexpected adaptive strategy in FLCR *C. parapsilosis*. Across outbreak isolates from multiple countries, we identify a paradoxical low-biofilm-producing (LBP) phenotype. Strikingly, biofilm attenuation correlates with enhanced host adaptation: LBP strains display increased immune evasion, resistance to macrophage and neutrophil killing, and improved survival in immune cell-rich organs during systemic infection. Transcriptomic profiling reveals extensive metabolic and regulatory rewiring in LBP strains, while whole-genome sequencing uncovers complex, multigenic alterations associated with biofilm attenuation. Disruption of key biofilm-regulatory transcription factors further enhances fitness during host immune interactions. Population genomic analyses demonstrate that the LBP phenotype has arisen independently across diverse outbreak lineages, consistent with convergent evolution under host immune selection.

Collectively, our findings challenge the long-standing paradigm that biofilm hyperproduction drives fungal outbreak persistence. Instead, we propose that *C. parapsilosis* undergoes an evolutionary tradeoff in which biofilm attenuation enhances immune evasion and promotes long-term persistence within healthcare-associated populations. These results redefine the ecological and evolutionary forces shaping fungal outbreak dynamics and highlight host-driven adaptation as a critical determinant of epidemic success.

## Results

### Fluconazole-resistant outbreak lineages exhibit attenuated biofilm formation

**Fig 1:**
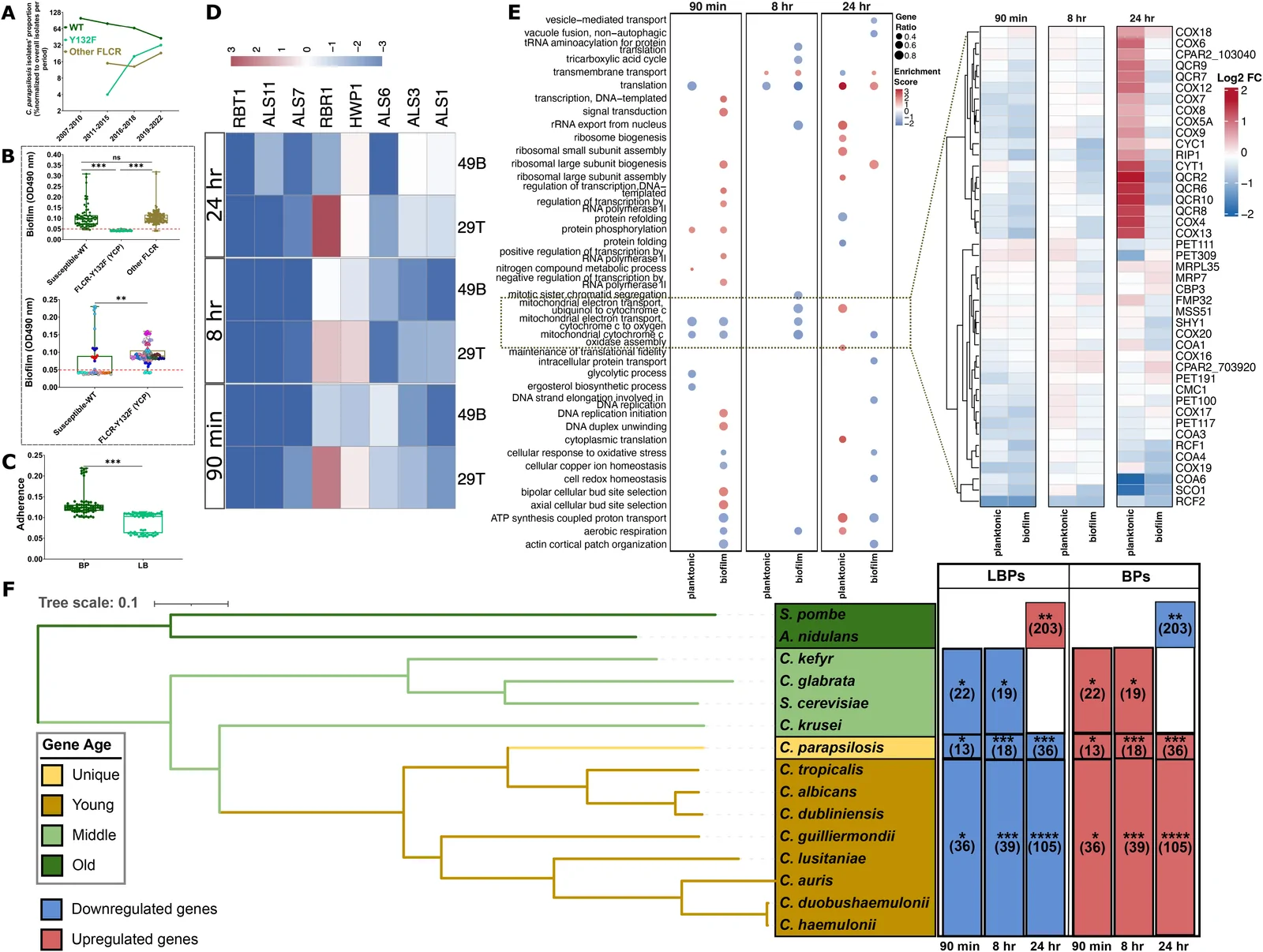
Trajectory of the evolution of *C. parapsilosis* outbreak in a Turkish hospital, biofilm formation ability of Turkish and Brazilian *C. parapsilosis* isolates, and RNAseq analysis of LBPs and BPs during various stages of biofilm. The prevalence of *C. parapsilosis* isolates collected from the bloodstream of patients from 2009-2024 identifies that fluconazole-resistant isolates carrying Erg11^Y132F^ progressively increase and outcompete the susceptible wild-type isolates **(A)**. Crystal violet biofilm analysis (OD_490_) revealed that Turkish *C. parapsilosis* isolates carrying Erg11^Y132F^ were LBP (upper), whereas the majority of the Brazilian (lower) *C. parapsilosis* isolates carrying Erg11^Y132F^ were BPs **(B)**. Since Brazilian YCP and WT isolates were a mixture of BP and LBPs, replicates belonging to the same isolate were color-coded to display the inter-replicate variability. LBPs from both countries showed an attenuated adherence during the early stage of biofilm formation **(C)**. Each dot represents an independent biological replicate. Genes pertinent for adhesion in *C. albicans* are downregulated in *C. parapsilosis* LBPs. Log_2_ fold change of gene expression in biofilm condition was obtained for LBPs, i.e., 49B and 29T, relative to BPs from their corresponding countries of origin (35B and 1T, respectively) **(D)**. Mitochondrial electron transport, cell redox homeostasis and aerobic respiration were exclusively upregulated in both LBP isolates at 24 h of biofilm formation. Genes belonging to the highlighted mitochondrial electron transport functions are downregulated in the biofilm condition in BPs and upregulated in LBPs. Gene names, when indicated, denote putative *S. cerevisiae* orthologs **(E)**. Log_2_ fold change of gene expression in biofilm and planktonic conditions were obtained for BPs (combining 35B and 1T) relative to LBPs (combining 49B and 29T) at each time point and gene set enrichment analysis was performed to obtain enriched biological processes **(E)**. Gene age was estimated and phylogenetic tree was constructed using orthologs across 15 species. “Old” genes are those conserved across *Ascomycota* species, “Middle” genes are conserved across CTG and non-CTG *Candida* species and *S. cerevisiae*, “Young” gene are conserved across CTG *Candida* species and “Unique” genes are those unique to *C. parapsilosis*. Genes upregulated at 24 h of biofilm formation in BPs (i.e., downregulated in LBPs) are enriched for “Young” and “Unique” genes, whereas genes upregulated at the same time point in LBPs are enriched for “Old” genes. A one-tailed hypergeometric test was conducted to statistically estimate the over-representation of each of these gene ages in differentially expressed genes and *p*-value was adjusted for multiple testing using Benjamini–Hochberg correction **(F)**. BP, biofilm producers; LBP, low biofilm producers. *, **, ***, and **** represents *P* values ≤0.05, ≤0.01, ≤0.001, ≤0.0001, respectively. Data underlying this figure could be found in the S1 Data file.

To define traits associated with outbreak persistence, we examined the longitudinal dynamics of bloodstream isolates of *C. parapsilosis* collected at Ege University Hospital (Turkey) between 2007 and 2024 [9,20,21]. During 2007–2010, all isolates were fluconazole-susceptible (FLCS). Over the subsequent decade, however, fluconazole-resistant (FLCR) isolates carrying the Erg11^Y132F^ substitution (YCP) progressively displaced the susceptible population and dominated by 2019–2022 (Fig 1A). The rapid and sustained expansion of this lineage suggested intrinsic fitness advantages beyond drug resistance.

Because similar Erg11^Y132F^ (YCP) outbreaks have been reported in Brazil, we assembled a collection of 120 isolates, comprising 95 from Turkey (historic outbreak) and 25 from Brazil (early-stage outbreak) (S1 Table). Given the long-standing assumption that biofilm formation promotes persistence in *Candida* species and that *C. parapsilosis* is shown to comparably produces biofilm relative to *C. albicans* [22], we quantified biofilm biomass using a validated high-throughput crystal violet assay. Of note, the crystal violet assay strongly and significantly correlated with other standard optical density measurements of biofilm quantification and with biofilm thickness measurements obtained by confocal scanning laser microscopy (S1A–S1C Fig), validating the approach. Based on this calibration, isolates with OD_490_ < 0.05 from the crystal violet assay were classified as low biofilm producers (LBP strains), whereas isolates with OD_490_ ≥ 0.05 were categorized as biofilm producers (BP strains) since those with OD_490_ < 0.05 remained transparent and did not show any crystal violet retention. Unexpectedly, all Turkish FLCR Erg11^Y132F^ isolates exhibited markedly reduced biofilm formation compared to both Brazilian FLCR isolates and FLCS isolates (Fig 1B). Whereas the majority of Brazilian isolates (“collected in early-stage outbreak”) retained robust biofilm capacity, Turkish outbreak isolates uniformly displayed a LBP phenotype. Additionally, while Turkish LBP isolates were all FLCR, the Brazilian counterparts encompassed a combination of FLCR and FLCS isolates (microbiological details are listed in S1 Table). Notably, impaired biofilm formation has also been reported in independent FLCR outbreaks in Brazil [23], Italy [24], and Spain [25], suggesting that biofilm attenuation is a recurrent feature of persistent lineages.

Consistent with impaired biofilm formation, LBP isolates exhibited significantly reduced surface adherence (Fig 1C). Pseudohyphal development, even under conditions known to favor pseudohyphal morphology in *C. parapsilosis* [26], was infrequent across all isolates and did not distinguish biofilm phenotypes.

Together, these findings challenge the prevailing view that enhanced biofilm formation underlies outbreak success and instead identify biofilm attenuation as a defining characteristic of dominant FLCR lineages.

### LBP strains exhibit metabolic rewiring and preferential activation of evolutionarily conserved transcriptional programs during biofilm development

The pronounced defect in biofilm formation among LBP isolates raised a fundamental question: how is their transcriptional program reconfigured during biofilm growth? To address this, we performed comparative RNA-seq to define the regulatory and evolutionary architecture underlying biofilm attenuation in outbreak-associated strains.

We selected one BP and one LBP isolate from each country: 29T and 49B (LBP, YCP lineage), and 1T and 35B (BP, FLCS lineage), where “T” and “B” denote Turkish and Brazilian origin, respectively. Because biofilm development proceeds through defined stages—early adhesion (90 min), initiation (8 h), and maturation (24 h)—transcriptomes were profiled at each time point under both biofilm and planktonic conditions.

Principal component analysis (PCA) revealed tight clustering of biological replicates, confirming high reproducibility (S1D Fig). Biofilm and planktonic transcriptomes diverged progressively over time, consistent with stage-specific differentiation (S1D Fig; S2A and S2B Table).

Given the pronounced adhesion defect in LBP strains, we first examined expression of established adhesin genes implicated in biofilm initiation. Consistent with the phenotype, most adhesins were downregulated in LBP isolates relative to BP isolates across all timepoints (Fig 1D). To define broader transcriptional trends, we normalized biofilm transcriptomes to their corresponding planktonic states. Strikingly, mitochondrial electron transport, aerobic respiration, and redox homeostasis were the only biological processes consistently upregulated in both LBP isolates at 24 h (S1E Fig). Reciprocal normalization of BP to LBP transcriptomes identified mitochondrial-associated processes as among the most significantly downregulated pathways across nearly all stages (Fig 1E). These data indicate that LBP strains activate a coordinated mitochondrial and oxidative metabolic program during biofilm growth, a pattern reminiscent of biofilm-defective transcription factor mutants in *C. parapsilosis* (*ace2*Δ, *bcr1*Δ, *cph2*Δ, *czf1*Δ, *gzf3*Δ, *efg1*Δ, and *ume6*Δ [27]).

Because biofilm regulatory networks in *C. albicans* are enriched for evolutionarily “young” genes unique to the CTG clade [28], we next asked whether evolutionary age distinguished transcriptional programs in BP and LBP isolates. Using comparative genomics across 15 yeast species (12 *Candida* and 3 non-*Candida* ascomycetes), we categorized genes into four age groups: “old” (conserved across all species), “middle” (shared among *Candida* species and *S. cerevisiae*), “young” (restricted to CTG *Candida* species), and “unique” (*C. parapsilosis*-specific).

Genes selectively upregulated in BP strains at 24 h were significantly enriched for “young” and “unique” genes, paralleling the lineage-specific innovation described for *C. albicans* biofilm networks [28]. In contrast, genes upregulated at 24 h in LBP strains were predominantly “old,” evolutionarily conserved genes associated with core metabolic functions (Fig 1F and S2C Table). Thus, whereas canonical biofilm formation relies on recently evolved, clade-restricted regulatory modules, the attenuated biofilm program of LBP strains preferentially engages ancestral metabolic circuitry.

Collectively, these findings indicate that outbreak-associated LBP isolates do not simply down-regulate biofilm genes; rather, they undergo a coordinated transcriptional reprogramming that shifts biofilm development away from lineage-specific adhesion networks and toward conserved metabolic processes.

### Whole-genome sequencing (WGS) reveals polygenic and convergent genetic architectures underlying biofilm attenuation

Biofilm impairment emerged as a defining feature of Turkish YCP isolates and a subset of Brazilian isolates. Because all Turkish YCP strains displayed the LBP phenotype, we initially hypothesized that acquisition of the Erg11^Y132F^ substitution directly impaired biofilm formation. However, CRISPR-Cas9-mediated reversion of Erg11^Y132F^ to WT Erg11 (F132Y) in two Turkish LBPs (59T and 60T) did not increase the biofilm capacity, whereas the revertant isolates showed 16- to 8-fold reduction in fluconazole minimum inhibitory concentration (S1F Fig), indicating that fluconazole susceptibility patterns and biofilm formation are decoupled and additional genetic determinants underlie the LBP phenotype.

**Fig 2:**
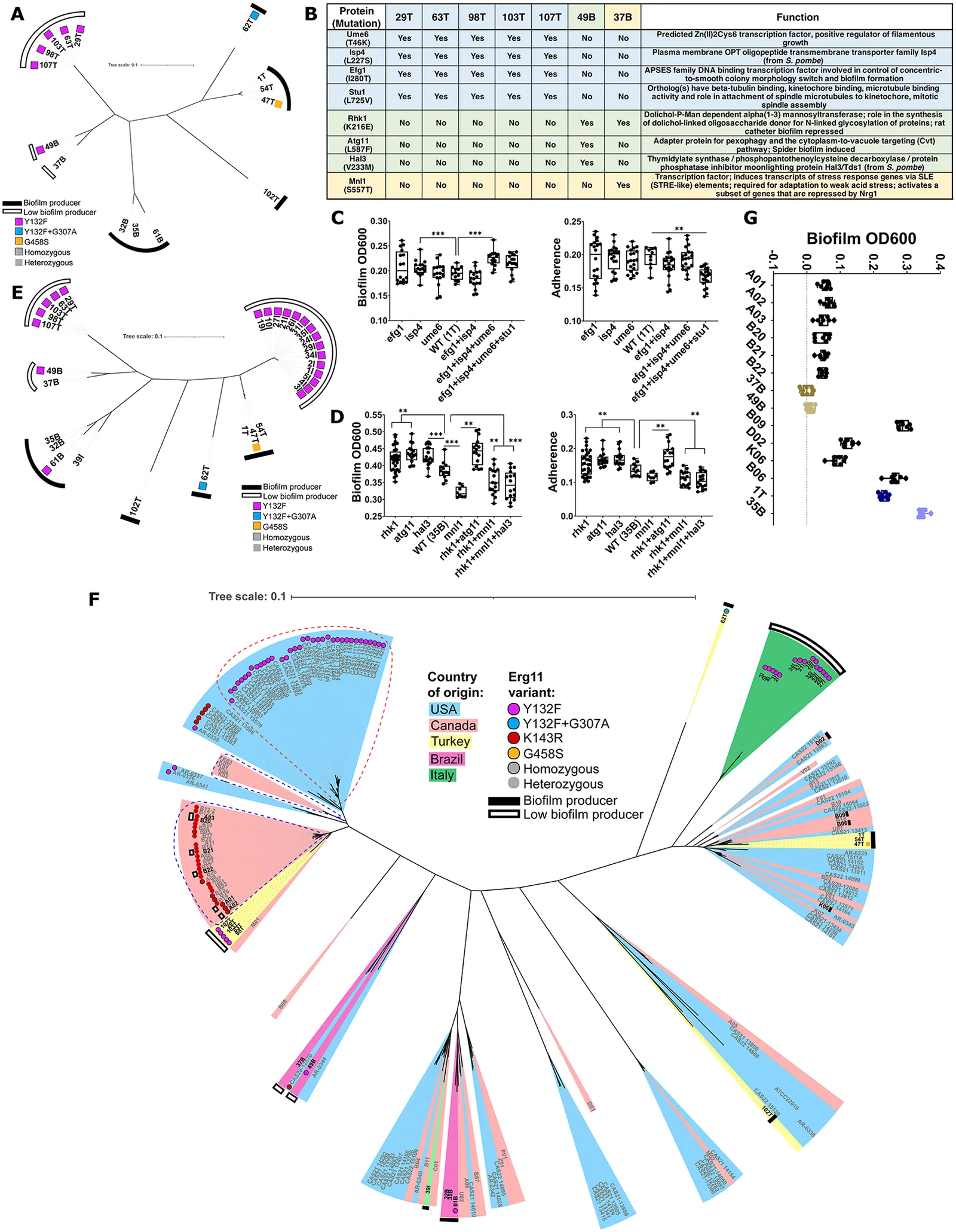
Whole-genome sequencing analysis and functional genomic studies to determine genomic structure and mutations underlying biofilm attenuation, respectively. Phylogenetic analysis of selected Turkish and Brazilian *C. parapsilosis* isolates **(A)**. Schematic presentation of mutations selected for genetic functional analysis among the Turkish and Brazilian LBPs **(B)**. Mutants were generated using CRISPR-Cas9 technology (Lombardi *and colleagues*, 2019), validated by PCR amplification and Sanger sequencing and at least 2–3 independent colonies were tested for each mutant. Biofilm (OD_600_) and adherence measured at 24 h and 90 min, respectively, for mutants created in the Turkish BP (1T) **(C)**. Biofilm (OD_600_) and adherence measured at 24 h and 90 min, respectively, for mutants created in the Brazilian BP (35B) **(D)**. Each dot represents an independent biological replicate for Fig A–D. Phylogenetic analysis of Italian, Turkish, and Brazilian *C. parapsilosis* isolates using maximum likelihood **(E)**. Phylogenetic analysis of global collection of *C. parapsilosis* isolates collected from outbreaks in multiple countries **(F)**. Canadian *C. parapsilosis* isolates clustered with Turkish LBPs (A01, A02, A03, B20, B21, and B22) produced a significantly lower biofilm (OD_600_) compared to counterparts grouped with Turkish BPs (B06, B09, D02, and K06) **(G)**. Dashed line indicates the lowest biofilm threshold. BP, biofilm producers; LBP, low biofilm producers. *, **, ***, and **** represents *P* values ≤0.05, ≤ 0.01, ≤ 0.001, ≤ 0.0001, respectively. Data underlying this figure could be found in the S1 Data file.

To define these determinants and assess the evolutionary relationships among isolates, we performed high-coverage (>100×) paired-end whole-genome sequencing of 16 Turkish and Brazilian strains. Isolates were selected based on biofilm formation capacity, susceptibility profiles, and *ERG11* mutations to account for various phenotypic and potentially genotypic differences. Phylogenetic reconstruction revealed that LBP isolates clustered together, forming two country-specific subclades, whereas BP isolates were more genetically diverse (Fig 2A). Unexpectedly, Brazilian LBP isolates were genetically closer to Turkish LBP isolates (2059.1 ± 57.3 mean SNP difference) than to Brazilian BP isolates (2862.8 ± 146.0 mean SNP difference), suggesting convergent or shared evolutionary trajectories associated with biofilm attenuation.

Notably, the Brazilian LBP clade included both YCP and FLCS isolates, whereas the Turkish LBP cluster consisted exclusively of YCP strains. We first explored if there exist phenotype-specific (LBP- or BP-specific) large or small chromosomal abnormalities. Our coverage analysis indicates that strain 1T has aneuploidy of chromosomes 4 and 8, having three copies of each rather than the typical two (S3A Table). Similarly, strain 47T has three copies of chromosome 6 (S3A Table). Several strains show evidence of previously discovered gene amplifications; including genes such as *RTA3* (CPAR2_104610), *ARR3* (CPAR2_601050), *CDR1B* (CPAR2_304370), and *ERG11*(CPAR2_303740) (S3B Table). Other genes have been deleted to one or zero copies in several strains (S3B Table). There are no genes that are amplified or deleted in all LBP strains or in all BP strains (S3B Table). Analysis of non-synonymous, stop-gain, and frameshift variants uniquely enriched in LBP isolates revealed that Turkish YCP strains shared a highly similar mutational profile, consistent with clonal expansion from a common ancestor. Gene ontology analysis identified enrichment in genes associated with ATP binding, purine binding, carbohydrate derivative binding, and ATPase activity (S2A Fig; S3C Table), although no single pathway was universally enriched across both countries.

To functionally interrogate candidate determinants of biofilm attenuation, we introduced prioritized LBP-associated mutations into BP backgrounds using CRISPR-Cas9 (Turkish mutations into 1T; Brazilian mutations into 35B). Given prior evidence that biofilm defects in *Candida* species frequently involve transcriptional regulators [18,19], we prioritized mutations in predicted regulatory genes, including Efg1^I280T^ and Ume6^T46K^, as well as additional candidates implicated by transcriptomic analysis (Fig 2B; S3D–S3J Table). Single, combinatorial (double, triple), and quadruple mutants were generated and sequence validated. Biofilm biomass and adhesion were quantified from two independent transformants per genotype relative to the wildtype (WT) (Fig 2B–2D).

None of the individual Turkish candidate mutations reduced biofilm formation. However, the combination of prioritized mutations resulted in a marked reduction in adhesion (Fig 2C), indicating that biofilm attenuation in Turkish LBP isolates likely arises through cooperative, polygenic effects rather than a single dominant mutation.

In contrast, analysis of a Brazilian LBP isolate (37B) identified a single mutation with strong phenotypic impact: Mnl1^S557T^. Introduction of this variant into a BP background substantially reduced biofilm formation and modestly decreased adhesion (Fig 2D). Introduction of Mnl1^S557T^ into the Turkish BP strain (1T) also produced a significant, albeit smaller, reduction in biofilm formation (S2B Fig). However, reversion of S557T to wildtype (T557S) in the 35B background did not restore biofilm formation (S2C Fig), suggesting that additional genetic context contributes to the phenotype. Indeed, whole-genome sequencing analysis revealed that 35B carries 23 heterozygous mutations (Compared to CDC317) in the region 685068-897009, all of which undergone loss-of-heterozygosity in Mnl1^S557T^ and retained in Mnl1^T557S^ (S4 Table). Contribution of those mutations in biofilm formation warrants future studies.

Expanded analysis of global *C. parapsilosis* genomes identified the same Mnl1^S557T^ variant in isolates from the United States (CAS2012570) [8] and Germany (GL28) [29], and revealed that *MNL1* polymorphisms are common across both BP and LBP clades (S2D Fig). Importantly, several BP isolates harbored N-terminal Mnl1 mutations, including an early stop codon (Q5*) present in Brazilian BP strains (32B, 35B, and 61B), indicating that simple loss of Mnl1 function is insufficient to confer biofilm attenuation. Moreover, ~37% of American FLCR isolates clustering with Turkish LBPs carried an alternative variant (Mnl1^V563I^), further supporting a complex and context-dependent role for Mnl1.

Collectively, these findings demonstrate that biofilm attenuation in outbreak-associated *C. parapsilosis* strains is not attributable to a single resistance mutation but instead arises through distinct, and in some cases polygenic, genetic architectures. The closer phylogenetic relatedness of LBP isolates across countries, relative to sympatric BP strains, further suggests that biofilm attenuation represents a recurrent adaptive trajectory during outbreak evolution rather than a geographically restricted event.

### Biofilm attenuation has emerged independently multiple times across the global *C. parapsilosis* population

To determine whether biofilm attenuation represents a geographically restricted phenomenon or a recurrent evolutionary trajectory, we expanded our comparative genomic analysis to include isolates with/without publicly available WGS datasets from additional outbreak-associated *C. parapsilosis* collections.

A recent Italian study reported that the majority of fluconazole-resistant isolates carrying Erg11^Y132F^ exhibited impaired biofilm formation [24]. We therefore selected 16 isolates from this cohort (15 FLCR LBP strains and one FLCS BP strain) for WGS and phylogenetic analysis. All Italian LBP isolates formed a tightly related cluster that was highly divergent from Turkish and Brazilian LBP clades (Fig 2E). In contrast, the single Italian BP isolate shared recent ancestry with Brazilian BP strains. Within-country SNP diversity was lowest among Turkish LBPs (mean 51.9 SNPs), intermediate among Italian LBPs (mean 103.8 SNPs), and substantially higher among Brazilian LBPs (936 SNPs), consistent with differing degrees of clonal expansion. Although multiple mutations were shared among Italian LBP strains, no specific biological pathways were significantly enriched (S3D–S3J Table), further suggesting that biofilm attenuation does not arise from a single conserved genetic module.

We next incorporated WGS datasets from outbreak-associated isolates from the United States [8] and Canada [7]. Because biofilm phenotypes were not available for these collections, we examined their phylogenetic relationships relative to our Turkish, Brazilian, and Italian isolates. BP strains from Turkey, Brazil, and Italy segregated into multiple distinct clusters, with the largest cluster comprising Turkish BP strains intermingled with several Canadian and American isolates (Fig 2F). In contrast, LBP strains formed three major clades: (i) a large cluster of Turkish LBP strains that included 48.2% of Canadian isolates carrying Erg11^K143R^ and 42.8% of American isolates branching from the same lineage; (ii) a smaller cluster in which 2.1% of American isolates grouped with Brazilian LBP strains; and (iii) a distinct Italian LBP clade that showed no close association with other global isolates. The close genetic relatedness between Turkish LBP strains and a subset of Canadian isolates prompted us to experimentally assess their biofilm phenotypes. Canadian isolates clustering with Turkish LBPs (A01, A02, A03, B20, B21, and B22) exhibited impaired biofilm formation, whereas isolates clustering with BP strains (B06, B09, D02, and K06) retained robust biofilm production (Fig 2G). These data demonstrate that phylogenetic clustering strongly predicts biofilm phenotype across geographically distinct collections.

Our global comparative analysis supports two non-mutually exclusive routes for the emergence of LBP strains: first, dissemination of ancestral LBP lineages across countries, as exemplified by Turkish LBPs clustering with Canadian and some American isolates, reminiscent of the global clade structure described for *C. auris* clades [30]; and second, independent, recent clonal expansion of biofilm-attenuated genotypes, as observed in Italy. Together, these findings indicate that biofilm attenuation is not an isolated regional anomaly but rather a recurrent and convergent feature of outbreak evolution in *C. parapsilosis*.

### LBP strains exhibit enhanced stress tolerance and cell wall remodeling

Loss of filamentation and biofilm capacity can, in certain contexts, confer a selective advantage. In *C. albicans*, gut-evolved isolates harboring loss-of-function mutations in biofilm- or filamentation-associated regulators display increased gastrointestinal fitness [19]. Similarly, *C. albicans eed1*Δ mutants, defective in filamentation and biofilm formation, exhibit elevated burdens during systemic infection [31] and oral colonization [32] compared to filament-competent strains. Notably, these biofilm-deficient isolates share features of metabolic reprogramming, consistent with our RNA-seq data indicating mitochondrial and oxidative metabolic remodeling in LBP strains, both of which are advantageous during interaction with innate immune cells [33–35].

Given the epidemiological pattern of persistent outbreaks—where a single genotype circulates across multiple patients—we hypothesized that biofilm attenuation may be coupled to enhanced fitness under host-relevant stress conditions.

**Fig 3:**
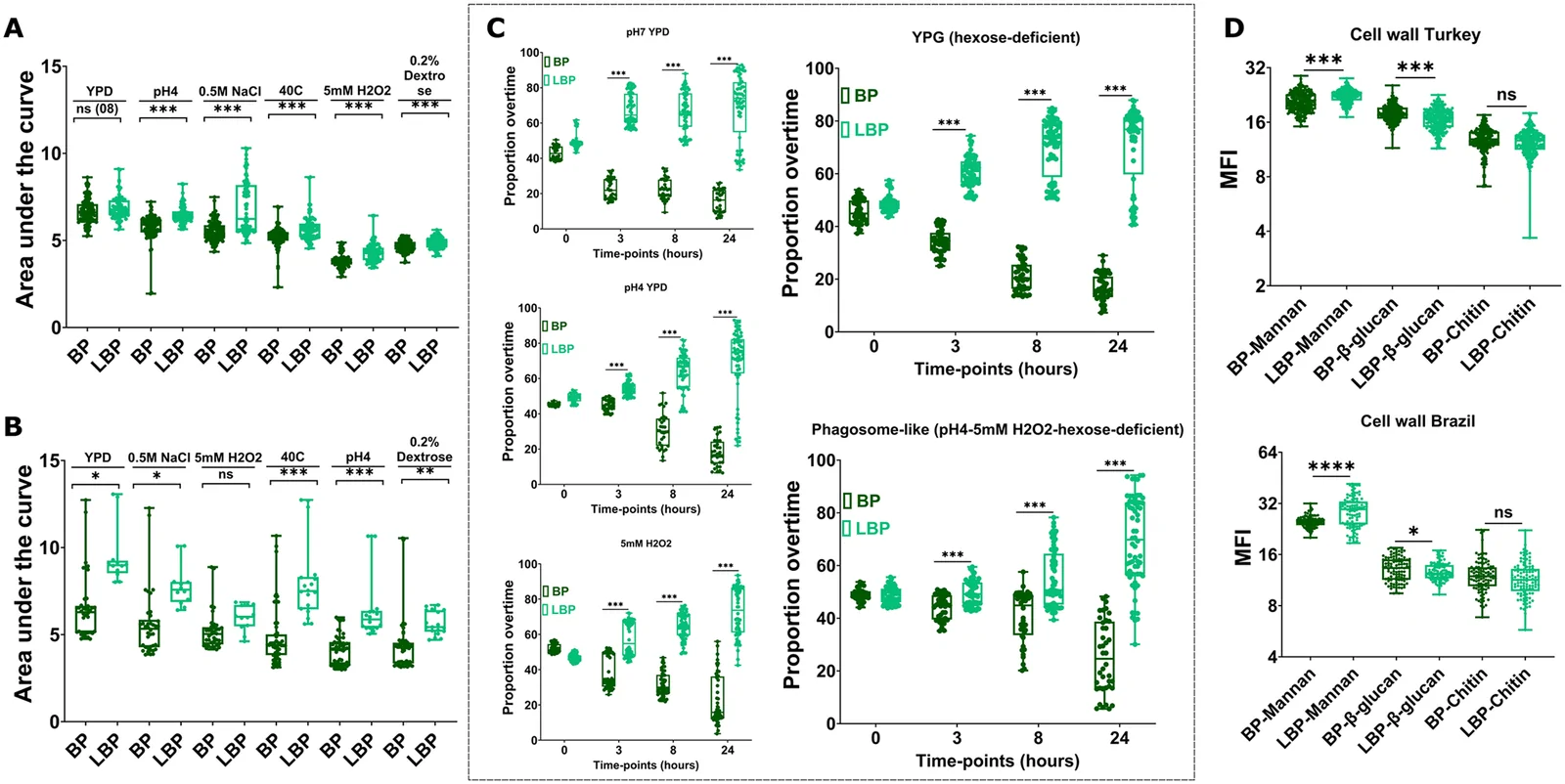
In vitro growth, stress tolerance, and cell wall exposure analysis of Turkish and Brazilian *C. parapsilosis* strains. AUC analysis under various conditions unravels that LBPs from Turkey **(A)** and Brazil **(B)** show a higher growth rate with/without stress. Cell suspension from overnight-grown isolates in YPD were adjusted at 0.2 OD600 in desired media and growth analysis was measured on an hourly basis. YFP-expressing LBPs outcompete mTorquise-expressing BPs under multiple 1:1 in-vitro competition analysis as judged by flow cytometry **(C)**. Cell suspensions were obtained from overnight-grown isolates, 1:1 mixture of BP and LBP were prepared, and YFP and mTorquise population sizes were determined at desired timepoints (0, 3, 8, and 24 h) using flowcytometry. YFP and mTorquise-expressing isolates were generated using CRISPR-Cas9 technology (Nemeth *and colleagues*, 2025) and validated by both Southern blot, PCR amplification, and Sanger sequencing. Each dot represents an independent biological replicate for Fig A–C. Cell wall exposure analysis using fluorescence microscopy uncovers that LBPs have a significantly lower β-glucan, but a markedly higher mannan exposure **(D)**. MFI of 50 fields were captured for each isolate and used for cell wall exposure analysis. AUC, area under the curve; BP, biofilm producers; LBP, low biofilm producers; YFP, yellow fluorescence protein; YPD, Yeast extract-peptone-dextrose; YPG, yeast extract-peptone-glycerol; MFI, Mean fluorescence intensity. *, **, ***, and **** represents *P* values ≤0.05, ≤0.01, ≤0.001, ≤0.0001, respectively. Data underlying this figure could be found in the S1 Data file.

To test this, we quantified growth dynamics of all Turkish and Brazilian isolates (*n* = 120) across a panel of stress and nutrient conditions: rich medium (YPD), low glucose (0.2% dextrose), acidic pH (pH 4), oxidative stress (5 mM H_2_O_2_), osmotic stress (0.5 M NaCl), and elevated temperature (40 °C). Growth was measured across full curves and summarized as area under the curve (AUC), with at least two biological replicates per isolate. Across nearly all conditions—including both stress and non-stress environments—LBP strains exhibited significantly greater growth than BP strains, independent of geographic origin (Fig 3A and 3B). These data indicate that biofilm attenuation is associated with a generalized growth advantage rather than a condition-specific effect.

To directly assess competitive fitness, we performed pairwise in vitro competition assays. Representative LBP and BP strains from both countries (LBPs were not from same genotypes, see genomic section) were differentially labeled (YFP and mTurquoise) and co-cultured at a 1:1 ratio under multiple conditions, including fermentable and non-fermentable carbon sources (YPD, YPG), low glucose, oxidative stress, acidic pH, and combined stress (S2E Fig). Population frequencies were quantified over time by flow cytometry. Consistent with monoculture growth phenotypes, LBP strains consistently outcompeted BP strains under all tested conditions, with competitive divergence increasing over time (Fig 3C). These results demonstrate that LBP strains possess a robust and environment-independent fitness advantage in vitro.

Because biofilm regulatory networks frequently intersect with cell wall remodeling pathways [36,37], and cell wall architecture critically shapes host–fungus interactions [38], we next examined cell wall architecture in a subset of isolates (*n* = 16 from both countries). Using fluorescence microscopy, we quantified surface exposure of chitin (WGA-Alexa 680), β-glucan (anti-dectin-1-Alexa 488), and mannan (Concanavalin A–Texas Red). LBP strains displayed significantly increased mannan exposure and reduced β-glucan exposure relative to BP strains (Fig 3D). Reduced β-glucan exposure is particularly notable given its role as a major ligand for innate immune recognition.

These findings indicate that biofilm attenuation in outbreak-associated strains is coupled to enhanced stress tolerance, competitive growth, and altered cell wall architecture. Rather than representing a simple loss of virulence capacity, which is biofilm attenuation, the LBP phenotype appears to reflect a coordinated physiological reprogramming that may enhance survival under host-imposed pressures.

### LBP strains exhibit enhanced immunoevasion and increased tolerance to innate immune cell interactions

β-glucan is a dominant fungal pathogen-associated molecular pattern (PAMP) recognized by host pattern recognition receptors (PRRs), including dectin-1, on innate immune cells. Masking of β-glucan outer mannan layers represents a well-established immune evasion strategy in fungal pathogens [38]. Given that LBP strains display reduced β-glucan exposure and increased mannan surface abundance, we hypothesized that they would be less efficiently recognized and eliminated by primary human innate immune cells. Here, we defined immunoevasion as reduced recognition and phagocytosis by innate immune cells. We therefore examined interactions of representative Turkish and Brazilian LBP and BP isolates with primary human monocyte-derived macrophages and neutrophils (*n* = 16 isolates, unless otherwise indicated). The LBP isolates represented distinct genotypes, as described in the genomic analyses below.

For macrophage interactions, phagocytosis was quantified as intracellular colony-forming units (CFUs) at 1 h post-infection (normalized to input), and intracellular survival was assessed at subsequent timepoints relative to the 1 h internalized population. Reactive oxygen species (ROS) production was measured by dihydrorhodamine 123 fluorescence at 3 h post-infection. Macrophages from at least two independent donors were used, and extensive washing ensured that only intracellular yeast were quantified.

**Fig 4:**
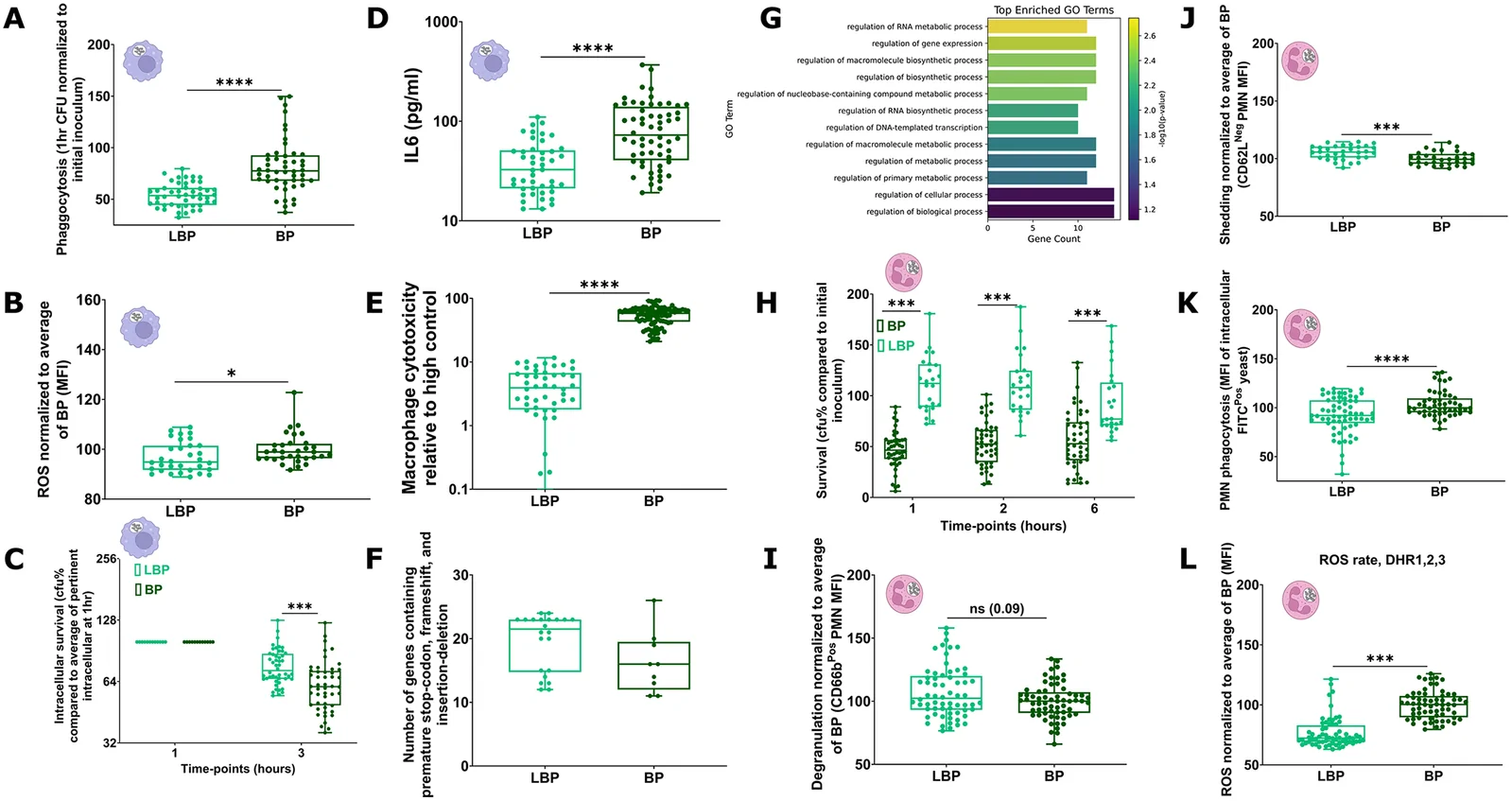
Interaction of *C. parapsilosis* strains with primary human macrophages and neutrophils. LBPs are immunoevasive and showed higher fitness during interaction with primary human macrophages and neutrophils. LBPs were less effectively phagocytosed by macrophages **(A)** and consistently macrophages infected with LBPs produced a lower ROS **(B)**. MFI of FITC-labeled yeasts phagocytosed by macrophages and DHR123 measured by flow cytometry were used for Fig A and **B.** Of note, ROS gating was adjusted based on un-infected control macrophages. Furthermore, intracellular LBPs showed a significantly higher survival (CFU) compared to intracellular BPs at 3 h (normalized to average of pertinent intracellular CFU at 1 **h) (C)**. Macrophages infected with LBPs produced a lower IL6 **(D)** and showed a lower damage as judged using lactate dehydrogenase assay **(E)**. Genomic analysis of Italian, Turkish, and Brazilian *C. parapsilosis* isolates showed that LBPs had a higher number of genes containing premature stop-codon, frameshift, and insertion and deletions **(F)** and those genes were mainly implicated in gene expression, biosynthetic, and metabolic processes **(G)**. Consistent with observations made with macrophages, neutrophils less effectively killed LBPs at all timepoints tested (CFU at each time point was normalized to pertinent initial inoculum) **(H)**, and neutrophils infected with LBPs showed a higher degranulation **(I)** and shedding (J) than those infected with BPs. Finally, LBPs were immunoevasive and less phagocytosed by neutrophils **(K)** and produced lower ROS compared to those infected with BPs **(L)**. Flow cytometry was used to determine the MFI of ROS, degranulation, and shedding for neutrophils infected with *C. parapsilosis* and gating was adjusted based on un-infected control neutrophils. Phagocytosis was determined by measuring the MFI of FITC-labeled yeasts phagocytosed by neutrophils using flow cytometry. Neutrophils and macrophages obtained from at least two healthy donors were used for analysis. Notably, phagocytosis was confirmed by extremely low association of *C. parapsilosis* cells with neutrophils and macrophages following cytochalasin D treatment. Each dot represents an independent biological replicate. BP, biofilm producers; LBP, low biofilm producers; ROS, reactive oxygen species; MFI, mean fluorescence intensity; CFU, Colony forming unit. *, **, ***, and **** represents *P* values ≤0.05, ≤ 0.01, ≤ 0.001, ≤ 0.0001, respectively. Created in BioRender. Löffler, J. (2026) https://BioRender.com/4kb4e7g. Data underlying this figure could be found in the S1 Data file.

LBP strains were significantly less efficiently phagocytosed than BP strains (Fig 4A) and elicited lower ROS production (Figs 4B and S2F). Notably the lower phagocytosis of LBPs could not be explained by their lower plastic adhesion, since (1) yeast-macrophage co-cultures were briefly centrifuged to bring the yeast cells in close proximity of macrophages, (2) the entire surface of the wells were covered by macrophages leaving limited space for attachment, and finally, (3) extensive washing steps and subsequent microscopic inspection ensured absence of extracellular yeast cells attached to the plastic. Between 1 and 3 h post-infection, LBP strains exhibited significantly greater intracellular survival relative to BP strains (Fig 4C), although survival equalized by 24 h (S2G Fig). Consistent with reduced β-glucan–dependent activation, macrophages infected with LBP strains (n = 35 isolates) produced significantly lower levels of IL-6 compared to BP strains at 24 h (Fig 4D), whereas other cytokines (TNFα and IL1β) were below detection in this assay.

We next assessed macrophage damage at 24 h using lactate dehydrogenase (LDH) release. LBP strains caused significantly less host cell damage than BP strains (Fig 4E), and consistently their extracellular CFUs at 24 h were lower compared to BP strains (S3A Fig). Together, these findings indicate that LBP strains are less cytotoxic, and more immunoevasive and tolerant of intracellular residence during early macrophage interaction.

Comparative genomic analysis further revealed that LBP strains harbored a trend towards a higher—though not statistically significant (*p* = 0.07)—number of genes containing premature stop codons or frameshift mutations relative to BP strains (Fig 4F and S3K Table). These mutations were enriched in genes associated with gene expression and metabolic processes (Fig 4G and S5 Table). While preliminary, this pattern is consistent with genetic streamlining observed in host-adapted bacterial pathogens [39,40] and supports the possibility that outbreak-associated LBP strains are undergoing specialization toward host-associated niches.

We next examined interactions with primary human neutrophils using the same isolate set. Survival was measured at 1, 2, and 6 h post-infection relative to input. Phagocytosis, ROS production, CD62L shedding, and CD66b degranulation were quantified by flow cytometry.

Across all timepoints, LBP strains exhibited significantly higher survival than BP strains (Fig 4H). Consistent with macrophage results, LBP strains were less efficiently phagocytosed (Figs 4K and S3D) and triggered significantly lower ROS production (Figs 4L and S3E). Although neutrophil degranulation was modestly increased in response to LBP strains, this difference did not reach statistical significance, whereas CD62L shedding was significantly elevated (Figs 4I–4J and S3B–S3C).

Collectively, these data demonstrate that LBP strains evade innate immune recognition, reduce inflammatory activation, and exhibit enhanced survival during interactions with both macrophages and neutrophils.

Rather than representing a simple loss of biofilm capacity, the LBP phenotype is coupled to coordinated cell wall remodeling and increased tolerance to immune-mediated stress. These features support a model in which biofilm attenuation is linked to enhanced host adaptation, potentially contributing to the persistence of outbreak-associated lineages.

### Macrophages infected with LBP strains adopt a proinflammatory transcriptional state and enhance early neutrophil fungicidal responses

The combination of reduced phagocytosis, diminished IL-6 production, limited macrophage damage, and enhanced early intracellular survival prompted us to examine the transcriptional response of macrophages to LBP (29T, 49B) versus BP (1T, 35B) strains at 3 and 24 h post-infection. Using the same isolates analyzed in fungal RNA-seq experiments, macrophages were extensively washed to remove extracellular yeast, lysed, and subjected to RNA-seq. Transcriptomes were normalized to uninfected controls.

Gene ontology (GO) analysis revealed distinct temporal patterns. At 3 h post-infection, macrophages infected with LBP strains were enriched for pathways associated with leukocyte cell–cell adhesion. In contrast, BP-infected macrophages showed strong enrichment at 24 h for granulocyte migration, neutrophil chemotaxis, and inflammatory signaling pathways (Figs 4B and S4A; S6 and S7 Tables).

**Fig 5:**
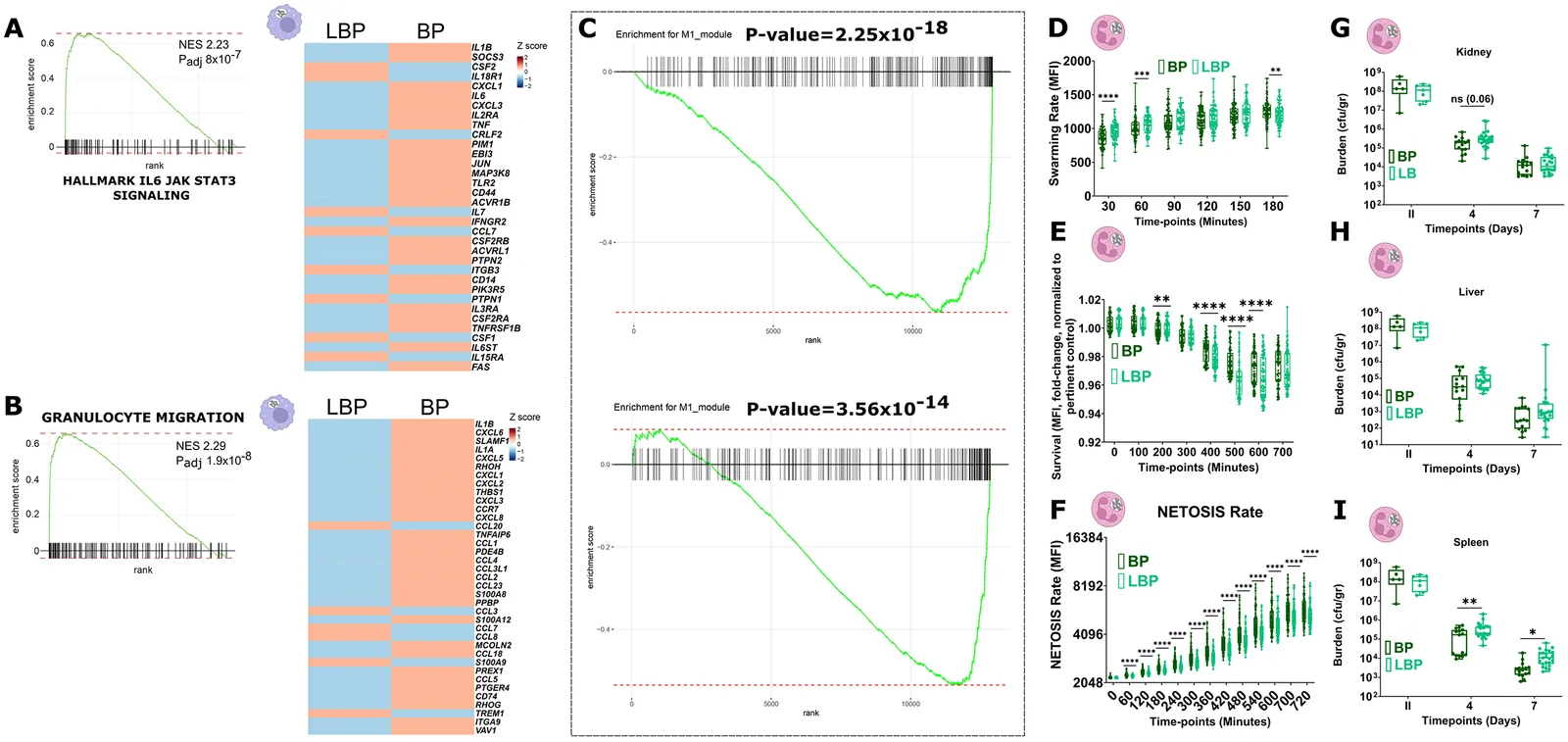
Macrophages infected with LBPs show a distinct transcriptional response and better induce fungicidal effector function of neutrophils. Enrichment plots depicting pathway enrichment (A, B: left) and heatmaps of transcript levels at the leading-edge for the “HALLMARK_IL6_JAK_STAT3_SIGNALING” pathway, and “GO:BP_GRANULOCYTE_MIGRATION” at 24 h (A, B: right). “Normalized enrichment score (NES)” and “adjusted *P*-value” are marked under the enrichment plots. Leading-edge analysis and heatmap showed that macrophages infected with the BPs, unlike LBPs, significantly upregulated the JAK-STAT signaling **(A)** and strong chemokine induction in the granulocyte neutrophil migration pathway **(B)**. Furthermore, macrophages infected with LBPs were skewed toward an M1 profile and were more proinflammatory at 3- and 24hrs post-infection **(C)**. Note that the *P* value is a nominal P-value, while NES is the normalized enrichment score **(C)**. Supernatants collected from macrophages infected with LBPs (at 24hrs post-infection), induced a higher swarming rate **(D)** and fungicidal effector function **(E)** of neutrophils toward immobilized iRFP-expressing *C. albicans* yeast cells, whereas neutrophils treated with supernatants of macrophages infected with BPs showed a higher NETOSIS rate **(F)**. Of note, supernatants were filter-sterilized using 0.2µ sterile syringe filters and MFI was used for swarming, fungicidal effector function (killing), and NETOSIS rates. Neutrophil fungicidal effector function (E) was determined by dividing the MFI iRFP-expressing *C. albicans* co-cultured with neutrophils over the MFI of pertinent *C. albicans* control at each time point. Each dot represents an independent biological replicate. LBPs showed comparable survival rate than BPs in kidney **(G)** and liver **(H)**, whereas they had a markedly higher survival rate in spleen compared to those infected with BPs **(I)** in the context of an intravenous (tail-vein injection) systemic infection mouse model. Mice were infected with 2*10^8^ CFU of LBPs and BPs and CFU were determined for each homogenized organ at designated timepoints by plating on YPD agar. Each dot represents an independent mouse. BP, biofilm producers; LBP, low biofilm producers; NETOSIS, neutrophil extracellular trap; CFU, Colony-forming unit; YPD, Yeast extract-peptone-dextrose; MFI, mean fluorescence intensity. *, **, ***, and **** represents *P* values ≤0.05, ≤0.01, ≤0.001, ≤0.0001, respectively. Created in BioRender. Löffler, J. (2026) https://BioRender.com/4kb4e7g. Data underlying this figure could be found in the S1 Data file.

GSEA provided further resolution. At 24 h, macrophages infected with BP strains exhibited coordinated upregulation of inflammatory mediators within the “Hallmark IL-6/JAK/STAT3” pathway (consistent with higher IL6 production of macrophages infected with BP strains), including *IL1B*, *IL6*, *TNF*, *CXCL1–3*, *SOCS3*, and *JUN* (Fig 5A and S8 Table), alongside robust induction of chemokines (*CXCL8*, *CCL2*, *CCL3*, *CCL4*, *CCL7*, *S100A8/A9*) associated with granulocyte recruitment (Fig 5B and S9 Table).

Previous dual species transcriptome studies demonstrated that in the initial stages of macrophage interactions with *C. albicans* yeast cells, the macrophages polarize toward a proinflammatory (M1-like) state, but at later time points, when *C. albicans* has filamented, the macrophages skew toward a less inflammatory (M2-like) state [41]. Since LBP strains exhibited hallmarks of intracellular containment—minimal macrophage damage and high survival between 1 and 3 h post-infection—we hypothesized that LBP strain-infected macrophages would exhibit an M1-like transcriptional state. Consistently, macrophages infected with LBP strains displayed a transcriptional profile enriched for genes classically associated with proinflammatory (M1-like) [41] activation at 24 h (Fig 5C and S10 Table). Notably, this occurred despite reduced cytokine release and diminished ROS induction, suggesting that LBP strains modulate the kinetics and nature of macrophage activation rather than globally suppressing inflammation. Therefore, despite reduced recognition and phagocytosis, intramacrophage LBPs tend to reprogram the immune responses toward an inflammatory state.

Because tissue-resident macrophages coordinate neutrophil recruitment and activation [42], we hypothesized that macrophage-derived mediators elicited by LBP strains would differentially influence neutrophil function. To test this, we used a microfluidic assay quantifying neutrophil swarming, fungicidal activity, and NETosis [43]. Filter-sterilized supernatants from macrophages infected for 24 h were added to neutrophil-*Candida* co-cultures, and responses were monitored by time-lapse confocal imaging.

Neutrophils exposed to supernatants from LBP-infected macrophages exhibited accelerated swarming dynamics (Fig 5D) and enhanced fungal killing (Figs 5E and S4C). In contrast, supernatants from BP-infected macrophages induced higher levels of NETosis (Fig 5F). These findings suggest that LBP strains promote a macrophage activation state that favors coordinated neutrophil recruitment and fungicidal activity rather than excessive NET formation.

Overall, these data reveal a nuanced immunological phenotype: although LBP strains are less efficiently recognized and killed during direct innate immune interactions, they induce a macrophage transcriptional program that enhances neutrophil-mediated fungal clearance. This pattern is consistent with a model in which outbreak-associated LBP strains balance immune evasion with sustained host engagement, potentially contributing to prolonged persistence under immune pressure.

### LBP strains exhibit enhanced persistence in immune cell-rich organs during systemic infection

Our in vitro and ex vivo analyses indicated that LBP strains possess a competitive growth advantage and enhanced tolerance to innate immune cell interactions. To determine whether these features translate into altered fitness during systemic infection, we evaluated representative Turkish and Brazilian LBP and BP isolates (*n* = 11 total strains, LBPs were not from the same genotype as shown in genomic section) in an immunocompetent murine model of hematogenously disseminated candidiasis.

Mice were infected via tail-vein injection with 2 × 10^7^ CFUs, and fungal burdens were quantified in kidneys, liver, and spleen at days 4 and 7 post-infection (3–4 mice per isolate per time point). Organs were homogenized and plated for CFU enumeration.

LBP strains exhibited modestly higher fungal burdens in the kidney and liver relative to BP strains, although these differences did not reach statistical significance (Fig 5G–5H). In contrast, LBP strains displayed a significantly higher fungal burden in the spleen (Fig 5I), an organ enriched in macrophages and other immune cell populations.

The preferential persistence of LBP strains in the spleen is notable given their enhanced resistance to macrophage and neutrophil-mediated killing observed ex vivo. Accordingly, LBP strains appear to display improved survival within immune cell-dense environments, consistent with their remodeled cell wall architecture and increased tolerance to innate immune stress.

Together, these findings indicate that biofilm attenuation is not associated with reduced in vivo fitness. Instead, outbreak-associated LBP strains demonstrate enhanced persistence in specific host niches, particularly immune cell-rich tissues, supporting a model in which biofilm loss is coupled to host-adaptive traits that promote survival during systemic infection.

### Disruption of biofilm regulatory circuits enhances survival during innate immune interactions

Our data thus far indicated that outbreak-associated LBP strains display enhanced survival during interactions with innate immune cells. We therefore hypothesized that attenuation of biofilm formation per se—whether arising through naturally occurring substitutions or targeted disruption of biofilm regulators—could promote fitness during host immune interactions. This hypothesis prompted us to assume that even random mutations leading to biofilm attenuation (such as Mnl1^S557T^) could also enhance fitness during interaction with innate immune cells.

**Fig 6:**
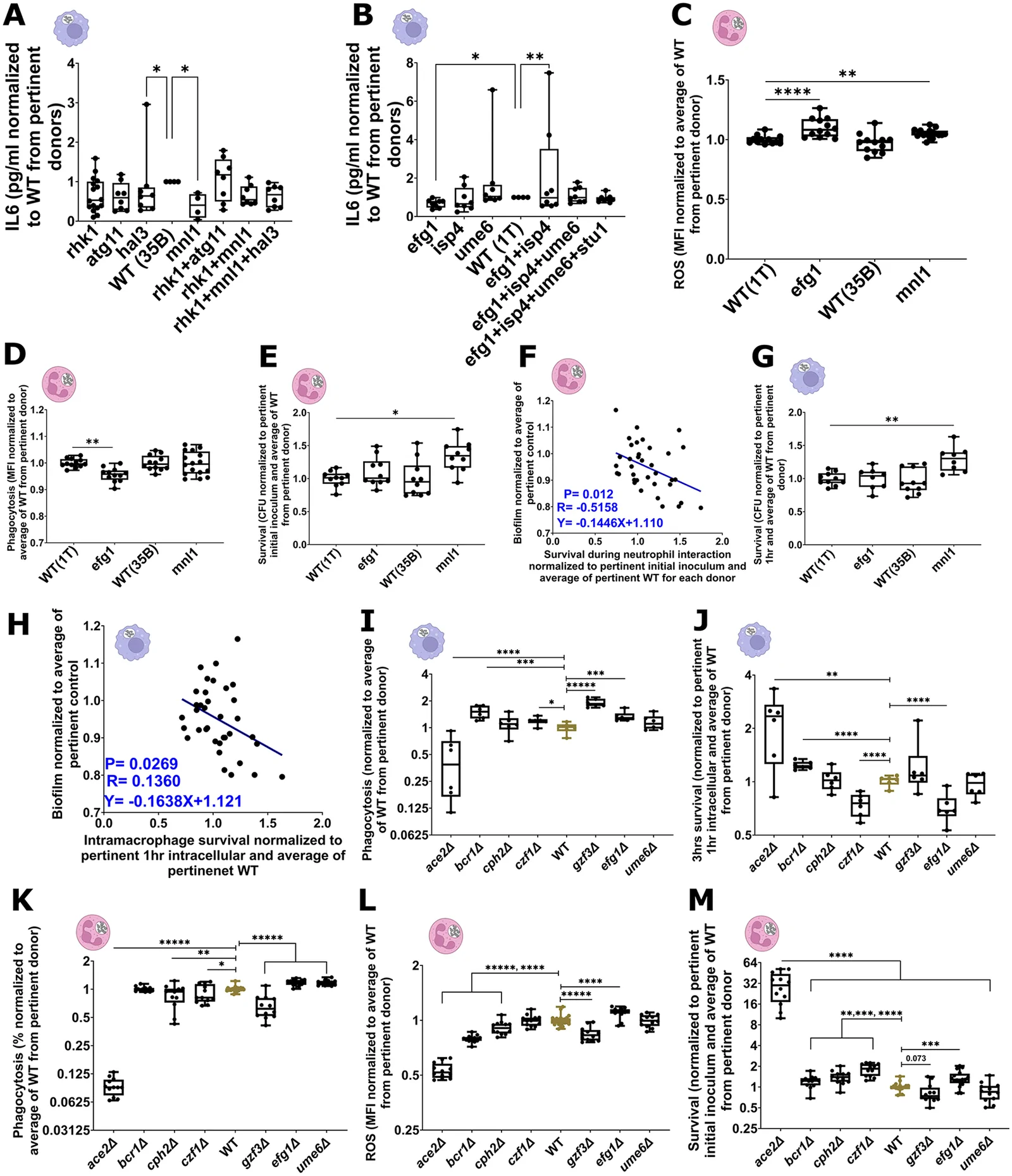
Biofilm formation of biofilm mutants created in Turkish and Brazilian BP backgrounds is negatively correlated with survival during interaction with primary human neutrophils and macrophages. Efg1^I280T^ **(A)** and Mnl1^S557T^ **(B)** induced lower IL6 production during interaction with macrophages. Neutrophils infected with mutants produced a higher ROS **(C)**, whereas only Efg1^I280T^ was less effectively phagocytosed by neutrophils **(D)** compared to pertinent wildtypes. Mnl1^S557T^ showed a higher survival during interaction with neutrophils **(E)** and biofilm level (OD_600_) of mutants was negatively correlated to survival during interaction with neutrophils **(F)**. Flow cytometry was used to determine the MFI of ROS for neutrophils infected with *C. parapsilosis* and gating was adjusted based on un-infected control neutrophils. Phagocytosis was determined by measuring the MFI of FITC-labeled yeasts phagocytosed by neutrophils using flow cytometry. Survival was determined by normalizing CFU at 2 h post-infection neutrophil against the CFU of the pertinent initial inoculum. All neutrophil effector functions obtained for each mutant were further normalized against the average of WT for each donor (to precisely capture how mutants impact neutrophil effector functions obtained from each donor and to minimize the donor-to-donor variability). Similarly, intracellular Mnl1^S557T^ had a higher survival rate during interaction with macrophages at 3 h post-infection (normalized to pertinent intracellular at 1 h post-infection) compared to pertinent wildtype **(G)** and biofilm level (OD_600_) was negatively correlated to intracellular survival at 3hr post-infection **(H)**. Phagocytosis **(I)** and survival **(J)** of *C. parapsilosis* mutants lacking key transcription factors/ kinase involved in biofilm regulation compared to parental WT strain (ATCC 22,019) during interaction with macrophages. Phagocytosis **(K)**, ROS **(L)**, and survival **(M)** of the same mutants of *C. parapsilosis* during interaction with neutrophils. *C. parapsilosis* survival during interaction with macrophages was determined by normalizing the CFU of intracellular yeast at 3 h against pertinent intracellular yeast CFUs at 1 h post-infection and data were further normalized to the average of WT for each donor (to precisely capture how mutants impact neutrophil effector functions obtained from each donor and to minimize the donor-to-donor variability). Neutrophils and macrophages obtained from at least two healthy donors were used for analysis. BP, biofilm producers; LBP, low biofilm producers; ROS, reactive oxygen species; MFI, Mean fluorescence intensity; WT, wildtype. *, **, ***, and **** represents *P* values ≤0.05, ≤ 0.01, ≤ 0.001, ≤ 0.0001, respectively. Created in BioRender. Löffler, **J.** (2026) https://BioRender.com/4kb4e7g. Data underlying this figure could be found in the S1 Data file.

To prioritize mutants for detailed ex vivo analysis, we first screened all strains generated in both Turkish and Brazilian isolates for IL-6 induction in macrophages, as LBP clinical isolates elicited markedly reduced IL-6 production. Macrophages infected with strains harboring Mnl1^S557T^ (Fig 6A) or Efg1^I280T^ (Fig 6B) produced significantly less IL-6 relative to their parental WT strains, consistent with the attenuated inflammatory phenotype observed in LBP isolates. These mutants were therefore selected for subsequent functional analyses.

Under stress-free and oxidative stress conditions (YPD, pH 4 YPD ± H_2_O_2_, YPG ± H_2_O_2_), neither mutant exhibited improved growth relative to WT (S4D and S4E Fig), suggesting that the enhanced stress tolerance observed in LBP clinical isolates likely requires additional mutations.

In contrast, clear differences emerged during immune cell interactions. Although neutrophils exposed to both mutants generated significantly higher reactive oxygen species (ROS) (Fig 6C), unlike clinical LBP isolates the Mnl1^S557T^ mutant demonstrated significantly enhanced survival despite similar phagocytosis rates (Fig 6D and 6E). This observation is expected as laboratory-generated mutants do not fully represent the complex genotype of clinical LBP isolates. Survival during neutrophil interaction negatively correlated with biofilm formation capacity (Fig 6F). Likewise, intracellular survival of the Mnl1^S557T^ mutant within macrophages was significantly increased relative to WT (Fig 6G), and macrophage survival similarly was inversely correlated with biofilm levels (Fig 6H).

These findings prompted us to test whether targeted deletion of established biofilm regulators in *C. parapsilosis* would recapitulate this phenotype. We examined *ace2*Δ, *bcr1*Δ, *cph2*Δ, *czf1*Δ, *gzf3*Δ, *efg1*Δ, and *ume6*Δ mutants, previously shown to regulate biofilm formation [25]. Reintegration of the WT alleles fully rescues biofilm formation in these strains [25], confirming that observed phenotypes are attributable to the targeted deletions. Consistent with prior reports and our in vitro validation (S4F Fig), *ace2*Δ, *bcr1*Δ, *czf1*Δ, *gzf3*Δ, *efg1*Δ, and *ume6*Δ exhibited attenuated biofilm formation, whereas *cph2*Δ displayed biofilm levels comparable to WT. Notably, all mutants showed reduced growth rates under standard and stress conditions (S4G–S4J Fig), indicating that any fitness advantages during immune interaction are unlikely to stem from generalized growth enhancement.

During macrophage interaction, phagocytosis rates were largely comparable to WT, except for *ace2*Δ, which showed reduced uptake (Fig 6I). Despite their growth defects, several biofilm-attenuated mutants exhibited enhanced intracellular survival: *ace2*Δ and *bcr1*Δ showed significantly increased survival, whereas *cph2*Δ, *gzf3*Δ, and *ume6*Δ were comparable to WT (Fig 6J).

During neutrophil interaction, *ace2*Δ and *gzf3*Δ elicited lower ROS production, consistent with reduced phagocytosis (Fig 6K and 6L). Importantly, most biofilm-attenuated mutants (*ace2*Δ, *bcr1*Δ, *czf1*Δ, and *efg1*Δ) demonstrated significantly enhanced survival following neutrophil exposure, while *gzf3*Δ and *ume6*Δ exhibited survival comparable to WT (Fig 6M).

Collectively, these functional analyses demonstrate that disruption of biofilm regulatory networks—whether through naturally occurring substitutions or targeted genetic deletion—does not impair, and frequently enhances, survival during innate immune interactions. These findings provide mechanistic support for an evolutionary trade-off model in which attenuation of biofilm formation is coupled to increased fitness within immune cell–rich host environments, thereby facilitating persistence during systemic infection and outbreak propagation.

## Discussion

By integrating multi-omics analyses, functional genetics, and extensive in vitro, ex vivo, and in vivo experiments, we demonstrate that impaired biofilm formation is a recurrent and globally conserved feature of recent *C. parapsilosis* outbreaks, enhancing tolerance to innate immune cell effector functions.

Our findings provide several novel microbiological insights. First, although biofilms serve as a robust protective shield against environmental stressors, including disinfectants, antifungals, and immune defenses, *C. parapsilosis* can attenuate the molecular networks underlying this ancient structure to better survive host immunity. This suggests that host pressures, rather than environmental stressors, are a major selective force shaping outbreak evolution. Supporting this notion, repeated gut passage of *C. albicans* favors mutants defective in filamentation and biofilm formation [19], and analogous patterns are observed in bacterial pathogens, where biofilm loss enhances host colonization or invasiveness, such as in African lineages of invasive nontyphoidal *Salmonella* (iNTS) [44,45].

A key question is why biofilm attenuation enhances in-host fitness. Our ex vivo macrophage studies indicate that LBP strains adopt a “yeast-locked intracellular lifestyle,” reminiscent of *C. glabrata*. LBP strains induce minimal macrophage damage, produce fewer extracellular yeasts at 24 h, and survive initial fungicidal activity more efficiently, correlating with higher spleen burdens in immunocompetent mice. Similar intracellular adaptation is seen in *Cryptococcus gattii* outbreak isolates, which exhibit enhanced macrophage fitness and virulence [46]. This yeast-locked lifestyle may facilitate bloodstream dissemination [31], conferring a selective advantage during systemic infection. Alternatively, biofilm formation may impose a metabolic cost within the host; impairing this pathway could conserve energy and enhance survival under immune pressure. Consistent with this concept, studies in *Salmonella* demonstrate that extracellular matrix components such as cellulose and curli fimbriae can act as PAMPs, and their loss improves infectivity [47–51]. These observations suggest that biofilm attenuation represents a conserved, *trans-*kingdom strategy to enhance microbial fitness against host immunity.

Second, the increased fitness of LBP strains may contribute to the higher mortality observed in patients infected with YCP isolates and to their persistence in hospital settings. Turkish patients infected with LBP strains exhibit significantly higher mortality compared to BP strain infections [9,20,21], likely reflecting enhanced survival within host tissues and immune evasion. While immunopathology may also contribute, these data suggest that LBP strains can persist independently of environmental biofilm reservoirs, highlighting human carriage (e.g., healthcare workers’ hands) as a major transmission route, reminiscent of *Salmonella Typhi* [52–54]. These findings underscore the need for infection control strategies targeting non-environmental reservoirs.

Third, our WGS analyses reveal that LBP strains can emerge from a shared ancestor or independently across distinct genotypes. Whether they originate from pre-existing biofilm-deficient isolates or evolve adaptively under host immune pressure remains unclear, but persistent immune selection clearly favors isolates capable of evading or resisting innate defenses.

Fourth, despite biofilm attenuation being a recently selected phenotype, LBP strains predominantly repurpose evolutionarily conserved genes for biofilm formation. In contrast, BP strains rely on younger or species-specific genes during late-stage biofilm development, consistent with prior observations in *C. albicans* [28]. This suggests that evolutionarily older genes can be flexibly repurposed to maintain minimal biofilm functions while supporting immune evasion.

Finally, although these findings may not directly generalize to other outbreak-causing pathogens such as *C. auris*, they caution against indiscriminate use of antibiofilm agents. Prolonged or repeated exposure to such compounds could inadvertently select for strains with enhanced immune evasion and systemic fitness. Investigating how antibiofilm interventions influence microbial adaptation and pathogenicity in host contexts is therefore warranted. Moreover, role of biofilm in outbreak persistence caused by other fungal pathogens, such as *C. auris* and *C. tropicalis*, requires future studies to further investigate if biofilm attenuation is *C. parapsilosis*-specific or generalizable to other fungal pathogens.

## Methods

### Ethics statement

This study was conducted in accordance with local legislation and institutional requirements, and pertinent protocols were approved by the ethics committee of Ege University Faculty of Medicine (20-2 T/30), University Hospital of Bari (Study 1338/CE- Approved Prot. 490 – 14/9/2023, “OUTBREAKS IN TERAPIA INTENSIVA: II valore della multidisciplinarietà tra Infection Control e Antimicrobial Stewardship), and part of a FAPESP GRANT (2017/02203-7) that was reviewed and approved by local Ethical Committee at Universidade Federal de São Paulo and Antimicrobial Resistance Institute of São Paulo.

### Kinetic growth analysis using plate reader and flow cytometry

*C. parapsilosis* isolates were grown in YPD overnight, followed by washing thrice with PBS, resuspending the OD600 of 0.2 in desired media, and dispensing 200 µl of cell suspensions in 96-well plates, sealed with Breathe-Easy^R^ sealing membrane covers (Millipore Sigma). Subsequently, plates were incubated at 37 °C, unless stated otherwise, in a Tecan Microplate Reader (Infinte 2000 pro) and OD600 reading with an hour interval. To capture the entire dynamic of growth curves, we measured the AUC for each replicate in all conditions.

For in-vitro competition experiments, we used YFP- and mTorquise-expressing LBP strains and BP strains, respectively. We created mixture of 1:1 of LBP strains and BP strains from different countries, inoculated pertinent media with 1:1 mixture, and monitored the proportion of each isolate in competition assays throughout the course of competition using a Celesta BD FACS Flow cytometer (BD Corporation).

### Biofilm assays

Biofilm assays using crystal violet, optical density, and confocal scanning laser microscopy assays were carried out as previously described [11,55]. Adherence assays were performed as previously described [56] except using OD600 as a readout.

### Cell wall staining of mannan and exposed chitin and glucan

*C. parapsilosis* isolates grown in YPD for 6 h (30 °C) were washed thrice with 1xPBS, followed by blocking with FACS block (0.5% BSA, 5% HI-rabbit serum, 5 mM ETDA, 2 mM NaAzide in PBS) for 30 min. Subsequently, pellets were washed thrice with FACS buffer (0.5% BSA, 5 mM EDTA, 2 mM NaAzide in PBS) at 4 °C, followed by resuspending the pellets in 5 µg/ml Fc:Dectin1 protein and incubated for 1 h at 4 °C. Next, pellets were washed thrice with PBS, stained with 50 µg/ml Wheat Germ Agglutinin-Alexa-680 to visualize exposed chitin, for 30 min at 4 °C. To visualize the exposed mannan, pellets washed thrice with PBS were stained with 50 µg/ml Concanavalin A-Texas Red for 30 min at 4 °C. DIC and fluorescence microscopy using an UltraVIEW VoX spinning disk confocal microscope (Nikon, Surrey, UK) were employed for all samples, and β1,3-glucan, chitin, and mannan exposures were measured by quantifying the MFI of each dye for at least 30 individual yeast cells of each isolate.

### Transformation of *C. parapsilosis*

Transformation involving the mutations found using WGS followed the exact CRISPR-Cas9 protocol described in our previous studies [57], whereas fluorescently labeled *C. parapsilosis* isolates were created in accordance with a previous study [58]. All oligonucleotides used for transformation purposes are listed in S11 Table.

### Macrophage isolation, infection, and functional analysis

Monocytes were sourced from leukopak of healthy donors attending Massachusetts General Hospital (IRB# 2014P002377). Following monolayer isolation using Ficol (ThermoFisher), monocytes were isolated using EasySep Direct Human Monocyte Isolation Kit (STEMCELL Technologies) in accordance with protocols described in our previous studies yielding high-quantity and quality monocytes [11,59]. Purity (CD45, CD16, CD14) and viability (staining with 7-AAD) were determined using flow cytometry, which were ≥93% and ≥99 in all experiments, respectively. Human macrophage-colony stimulating factor (hM-CSF, 50ng/ml) was used to differentiate monocytes into fully mature macrophages in fresh complete RPMI = cRPMI (10% heat-inactivated FBS, 1% pen-strep, 1% L-glutamine) as described previously. Overnight YPD-grown *C. parapsilosis* isolates washed thrice with PBS (at desired MOIs) were used to infect fully mature macrophages on day 8.

To examine the intracellular survival of *C. parapsilosis*, macrophages were infected with an MOI = 3/1, followed by incubation in at CO_2_ incubator for 1 h, extensively washing macrophages to remove the non-engulfed *C. parapsilosis* cells at 1 h pi, adding fresh cRPMI and returning to CO_2_ incubator. Macrophages were lysed with ice-cold water at designated timepoints (1-, 3-, and 24-h), followed by plating on YPD agar plates and incubating plates at 37ºC for 48h and counting CFUs. Phagocytosis was determined by normalizing the CFU of intracellular *C. parapsilosis* at 1 h pi against the pertinent initial inoculum, whereas survival was measured by normalizing the intracellular *C. parapsilosis* CFUs at designated timepoints against the pertinent 1 h intracellular *C. parapsilosis* CFUs. To measure ROS, macrophages were stained with CellMask-Deep Red (ThermoFisher, APC700) prior to infection, followed by infection with *C. parapsilosis* isolates (MOI = 10/1), washing macrophages 1h pi, adding dihydroxyrhodamine 1,2,3 (DH1,2,3, ThermoFisher) and incubation for an extra 2 h. Flow cytometry was used to determine the MFI of ROS of APC700^+^ infected macrophages. Un-infected macrophages were used for gating the ROS.

### Cytokine measurement

Primary human macrophages were infected with *C. parapsilosis* isolates at an MOI = 10. Supernatants were collected 24 h pi and subjected to cytokines were quantified using ELISA MAX Deluxe set Kits (BioLegend) in accordance with the manufacturer’s instructions [60].

### Macrophage cytotoxicity measurement

Macrophage cytotoxicity used a commercially available LDH kit (Sigma Aldrich) in accordance with a previously described protocol [61]. Macrophages were infected with the MOI of 8 yeasts/1 macrophages (MOI = 8/1), followed by extensively washing macrophages with PBS at 3 h pi. After an additional 21 h incubation in CO_2_ incubators, plates containing infected macrophages were centrifuged (1,500*g*, 5 min, room temperature), collecting supernatants, and subjecting them to LDH kit. Negative control included supernatant of un-infected macrophages, the value of which was subtracted from all infected counterparts, and positive control was un-infected macrophages treated with 0.25% Triton 100-X for 3 min. The subtracted value of all infected macrophages was normalized against positive control and data were presented as percentage.

### Neutrophil isolation and infection

EasySep Human Neutrophil Isolation Kit (STEMCELL Technologies) were used to isolate fresh neutrophils from the blood of healthy donors (IRB #2014P002377), which yields highly pure and viable neutrophils (≥99%). Phagocytosis, ROS production, degranulation, shedding (all MOI = 3/1), and *C. parapsilosis* survival (MOI = 1/3) were determined as outlined previously [11,62]. Neutrophil swarming and analysis were carried out in accordance with previously described protocols without modification [11]. To study the impact of macrophage supernatants on neutrophil activities, macrophages infected with LBP strains and BP strains for 24 h were subjected to centrifugation (500*g*, 5 min, room temperature), followed by collecting supernatants, adding active 10% FBS, passing though 0.2µ filter units and storing them in CO_2_ incubator until use. Sixteen-well microfluidic chambers containing iRFP-expressing *C. albicans* were mounted on confocal microscopy (Nikon Inverted Microscope Eclipse Ti-E equipped with a CSU-X1 confocal spinning disk head (Yokogawa)), followed by adding freshly isolated neutrophils treated with pertinent supernatants and measuring swarming rates, NETOSIS, and *C. albicans* viability (MFI of iRFP as a proxy for survival) and capturing images every 10 min for 12 h.

### RNA extraction, library preparation, and sequencing

Growth of *C. parapsilosis* biofilms was performed as described previously [22]. Biofilms were harvested by gently pipetting up and down along the bottoms of the 12-well plates and combining the biofilm slurry of the same strain from each well of one 12-well plate into a 50 mL conical tube. Planktonic cultures of a given biological replicate were combined in a 50 mL conical tube. Three biological replicates were completed for each sample and each time point. The conical tubes were centrifuged at 4,000*g* for 5 min and the supernatant was aspirated. Pellets were snap-frozen and stored at −80 °C until RNA extraction. Total RNA was extracted using the Ribopure RNA Purification Kit for Yeast (AM1926). mRNA was separated from total RNA using the NEBNext Poly(A) mRNA Isolation Module (E7490L). 500 ng of purified mRNA were made into cDNA libraries using the NEBNext Ultra II RNA Library Prep Kit for Illumina (E7775L). Libraries were sequenced on the AVITI 150 PE75 at the UC Davis Sequencing Core.

To assess the transcriptomic responses of macrophages, they were infected with *C. parapsilosis* (MOI = 5/1), subjected to extensive washing with PBS at 1 h post-infection, followed by lysing macrophages using a previously described RLT buffer-based protocol. Following DNA removal using RNase-free DNase, RNA samples were subjected to purification using RNeasy kit (QIAGEN) per manufacturer’s instruction. High-quality RNA samples determined by running RNA samples on 1.5% agarose gel and NanoDrop (ThermoFisher) were used for RNA-seq using mRNA capture as described in our previous studies. Library preparation and RNAseq were performed by Azenta Life Sciences in the exact same manner as described previously [63]. RNA-seq data of macrophages infected with *C. parapsilosis* can be accessed with the following GEO accession number GSE344550.

### RNA-Seq data processing for *C. parapsilosis* transcriptome

The paired-end reads were assessed for quality using FastQC (v0.11.9) [64]. The reference *C. parapsilosis* genome and its annotation were retrieved from the *Candida* Genome Database (CGD) [65] on 16th January, 2024. The reference genome was indexed using the subread-buildindex function in subread (v2.0.6) [66]. We then mapped the paired-end reads to the reference genome using the subread-align function in subread (v2.0.6) [66], including multi-mapped reads, mapping to the best 10 genomic loci (parameters: –multiMapping, -B 10). Transcript abundance for each gene was computed using the featureCounts function in subread (v2.0.6) [66], counting only read-pairs where both ends map to the same chomosome (parameters: -B, -C, –countReadPairs). The transcript abundance table was generated by consolidating all serial isolates, biological replicates, time point (90 min, 8 h and 24 h) and growth condition (biofilm and planktonic growth condition) using a custom Python script (v3.8.18) and were used for subsequent differential expression analyses.

### Differential expression and functional enrichment analysis for *C. parapsilosis* transcriptome

Transcript abundances were used to identify differentially expressed genes in the LBP strains (29T and 49B) relative to BP strains (1T and 35B) from the corresponding country of origin across three timepoints (90 min, 8 h and 24 h) in the biofilm condition. Additionally, strain-specific gene expression in biofilm condition was obtained relative to planktonic growth for each strain independently at each time point. Differential expression analysis was performed using DESeq2 (v1.44.0) [67]. P-value estimates were adjusted for multiple hypothesis testing using independent hypothesis weighting (IHW) (v1.32.0) [68] and log2 fold change shrinkage was performed using apeglm (v1.26.1) [69]. For the heatmap comparing expression of adhesion-related genes, we obtained genes in *C. albicans* involved in this process [70] and identified corresponding orthologs in *C. parapsilosis* as annotated in CGD [65]. The heatmaps were generated using the pheatmap package (v1.0.12) in R (v4.4.3). Functional enrichment of gene categories across isolates, was performed using GSEA [71] by sorting genes in their descending order of expression. GSEA was performed using the clusterProfiler package (v4.12.6) [72] in R (v4.4.3). Gene age enrichment was performed for the set of genes differentially expressed in both LBP strains relative to the corresponding BP from the corresponding country of origin. To infer gene age, orthologs of *C. parapsilosis* genes were identified across 8 CTG-clade species (*C. albicans*, *C. dubliniensis*, *C. tropicalis*, *C. guilliermondii*, *C. auris*, *C. haemulonii*, *C. duobushaemulonii*, *C. lusitaniae*), 3 non-CTG species (*C. glabrata*, *C. kefyr*, *C. krusei*) along with *Saccharomyces cerevisiae,* a close relative of *C. glabrata* and 2 distantly related Ascomycota species (*Aspergillus nidulans* and *Schizosaccharomyces pombe*) using OrthoFinder (v2.5.4) [73]. If a *C. parapsilosis* gene is found only in CTG-clade species, it is considered “Young”, if it is also found in non-CTG species it is considered “Middle” age and if it is found in distant Ascomycota species, it is considered “Old”. If no ortholog of the *C. parapsilosis* gene is found in any other CTG, non-CTG or Ascomycota species mentioned above, it is considered “Unique” to *C. parapsilosis.* To estimate enrichment of different gene ages for a set of genes, a hypergeometric test was conducted, and p-value was adjusted for multiple testing using Benjamini-Hochberg correction. RNAseq data of *C. parapsilosis* isolates under planktonic and biofilm conditions are accessible using the following GEO accession number GSE312575.

### Bulk RNA-Seq data processing and enrichment analysis for macrophages’ transcriptome

Raw counts from RNA-seq data and the associated sample metadata were loaded in R and harmonized by concatenating “Sample.name”, “group”, and “time point” to align column names between matrices. *C. parapsilosis* isolates were stratified into BP and LBP groups (BP: 1T, 35B; LBP: 29T, 49B) and timepoints (control, 3 h, 24 h). Data were analyzed using edgeR [74]; briefly, library sizes were normalized, lowly expressed genes were filtered, and a design matrix without an intercept was built on the interaction of biofilm group and time (model: ~ 0 + group:time). Differential gene expressions were tested for macrophages stimulated with “BP” versus “LBP” isolates at 3 and 24 h post-infection. Significant differentially expressed genes were defined as genes showing with |log2FC| > 1. For assessing biological pathway enrichment, we used fast geneset enrichment analysis (fgsea) on preranked gene lists (ranks = log2FC for BP-LBP) for both 3 and 24 h. Gene sets included MSigDB Hallmark (h), Canonical pathways (C2), Gene Ontology (C5), and a curated macrophage M1 versus M2 polarization geneset [71,75,76]; GSEA was run with minSize = 15 and maxSize = 500. Enrichment curves and top pathways were plotted directly from plotEnrichment. To interrogate biology of specific pathways (e.g., HALLMARK_IL6_JAK_STAT3_SIGNALING, GOBP_GRANULOCYTE_MIGRATION), leading-edge (LE) genes were extracted from the fgsea results and their group means (LBP STRAINS versus BP) at the target time point were computed from log2(CPM + 1). For visualization, heatmaps were generated with ComplexHeatmap, where rows were ordered by contribution to the observed Normalized Enrichment Score –NES (using the NES sign and the BP-LBP ranks), columns were fixed as LBP or BP (without clustering), and colors reflected row *Z*-scores to emphasize relative induction.

### Alignment and variant Calling

Short sequencing reads were trimmed and filtered using Skewer v0.2.2 using parameters “-q 30 -l 35” [77]. The resulting trimmed reads were assessed for quality by FastQC (https://www.bioinformatics.babraham.ac.uk/projects/fastqc/). Reads were mapped onto the *C. parapsilosis* CDC317 reference genome (GCA_000182765.2), using the Burrow–Wheeler Aligner (BWA-MEM) algorithm version 0.7.17 [78]. The aligned reads were marked for duplicates using the GATK version 4.2.0.0 MarkDuplicates tool and reindexed using GATK’s BuildBamIndex tool [79]. To produce highly accurate variants, thee separate variant calling tools were used in parallel and the results combined. Variants were called using bcftools mpileup version 1.10.2 [80], and FreeBayes version 1.3.9 [81] with standard parameters. Variants were also called with GATK using the tools HaplotypeCaller, CombineGVCFs and GenotypeGVCFs. The thee resulting files were merged using bcftools isec [79], keeping only variants that were called by at least two of the thee tools. Variants were filtered using GATK VariantFiltration [79] to remove variants with genotype quality <70 and read depth <20. Additionally, SNPs that were present in clusters of 5 or more within 20 bp were removed as artifacts, and only biallelic variants were kept.

### Copy number variation analysis

Copy number was estimated for all *C. parapsilosis* genes using the short-read alignments. The mean coverage across the gene’s open reading frame was found using BEDTools coverage version 2.29.2 [82]. This was normalized by dividing by the modal genome coverage as found using BEDTools genomecov [82] and then multiplying by two to account for ploidy. The data were filtered to keep only genes that had an abnormal copy number, i.e., greater than 2.75× or less than 1.25×, in at least one strain. Chromosome ploidy was used by calculating mean coverage across the entire chromosomes and normalized as above.

### Functional analysis

SnpEff was used to annotate the final set of variants for their predicted effect [83]. A subset of variants including only those likely to affect protein function (missense mutations, nonsense mutations, frameshifts, etc.) as input for functional GO analysis. GO analysis was performed using the Candida Genome Database website (http://www.candidagenome.org/). Orthologs of *C. parapsilosis* genes in *Candida albicans* and *Saccharomyces cerevisiae* were identified using CGOB [84]. Protein domains and families were predicted using InterPro [85].

### Phylogenetics

For phylogenetic analysis, indels were removed to keep only SNPs. A custom script was used (https://github.com/CMOTsean/HetSiteRando) to create a FASTA alignment of all sites in the genome that had a SNP in at least one sample. Heterozygous sites were randomly resolved to either the reference or alternate allele. 12,199 SNPs were used to construct the phylogeny of strains sequenced in this study, and a total of 33,534 SNPs were used in the alignment for the phylogeny including strains from other studies. RAxML version 8.2.12 [86] was used to construct a tree from this FASTA alignment using the GTRGAMMA model of nucleotide substitution with 1,000 bootstrap replicates. Trees were visualized using the iTOL website [87] and manually annotated.

### Whole-genome sequencing

DNA was fragmented to sizes between 1 and 20 kb using a transposase that binds biotinylated adapters at the breaking point. Strand displacement was performed to “repair” the nicks left by the transposase. Fragment sizes of 3–6 kb were then selected on a 0.8% agarose gel and were then circularized. Non-circularized DNA was removed by digestion. The circular DNA was then mechanically sheared to fragments of 100 bp to 1 kb approx. and the fragments containing the biotinylated ends were pulled down using magnetic streptavidin beads and submitted to a standard library preparation. A final size selection on 2% agarose gel was done and fragments of 400–700 bp were selected for the final library. Final libraries were analyzed using Agilent High Sensitivity chip to estimate the quantity and check size distribution and were then quantified by qPCR using the KAPA Library Quantification Kit (ref. KK4835, KapaBiosystems) prior to amplification with Illumina’s cBot. Libraries were sequenced 2 × 150 bp on an Illumina’s NovaSeq instrument. Raw sequencing reads have been deposited at Short Read Archive (PRJNA1363747).

### Systemic infection mouse models

Systemic infection mouse models were carried out according to the previously established protocol (IACUC protocol #2017N000058) [11]. Briefly, mice were infected with 2*10^7^ CFU of each isolate using tail vein injection and sacrificed on designated timepoints, followed by harvesting and homogenizing organs and plating on YPD agar plates for CFU counting. The CFUs obtained from each organ were normalized against the CFUs of the pertinent initial inoculum used to infect mice. For experiments involving antifungal treatment, mice received desired antifungal drugs 24 h post-infection and treated every other day. CFUs of organs at desired time point were normalized against the pertinent untreated control mice and results were presented as percentages.

### Statistical analysis

SPSS software (v.24 for Windows; SPSS, Chicago, IL, USA) was used in our statistical analysis and statistical significance was designated for *P*-values ≤0.05. We used Shapiro–Wilk test to determine data distribution and Wilcoxon test was used to determine statistical significance of nonparametric data.

## Supporting information

S1 Fig Crystal violet assay was positively correlated with standard biofilm quantification methods. (OD_600_) **(A)** and confocal scanning laser microscopy **(B, C)**. PCA **(D)** and GO terminology enrichment analysis **(E)**. Upper figure shows the biofilm level of 59T and 60T (fluconazole-resistant, carrying Erg11^Y132F^, and low biofilm producer) and revertant colonies (fluconazole susceptible and carrying wild-type Erg11^F132Y^) determined by crystal violet (OD490) and lower figure displays the fluconazole susceptibility pattern (µg/ml) of those isolates **(F)**. The data underlying this S1D and S1E Fig can be found in https://doi.org/10.5281/zenodo.21799493 and the rest in the S1 Data File.
(TIFF)

S2 Fig Mutations exclusive to low biofilm producers (LBP) were enriched for genes belonging to diverse biological functions. **(A)** Introduction of Mnl1^S557T^ into a Turkish LBP (1T) decreased the biofilm formation **(B)**, but its reversion of Mnl1^S557T^ to WT did not restore the biofilm defect **(C)**. Whole-genome sequence analysis of global collection of *C. parapsilosis* isolates identified various MNL1 mutations in numerous *C. parapsilosis* isolates from a global collection **(D)**. Flow cytometry gating strategies using mTorquise and YFP expressing *C. parapsilosis* isolates **(E)**. Flow cytometry gating strategies used to measure the reactive oxygen species (ROS) of macrophages **(F)** infected with *C. parapsilosis*. Gating strategy was drawn based on the uninfected control macrophages and mean fluorescence intensity was recorded. Macrophages infected with biofilm producers and LBPs showed similar intracellular growth rates at 24 h post-infection **(G)**. The intracellular colony-forming units (CFUs) at each time point were normalized against the average of pertinent 1 h intracellular CFUs. The data underlying S2E and S2F Fig can be found in https://doi.org/10.5281/zenodo.21794379 and the rest in the S1 Data File.
(TIFF)

S3 Fig Flow cytometry gating strategies used to determine the neutrophil effector functions against *C. parapsilosis* isolates. Low biofilm producer *C. parapsilosis* isolates showed a significantly lower extracellular colony-forming units (CFUs) relative to biofilm producers at 24 h post-infection of macrophages **(A)**. Flow cytometry gating strategies used to measure the degranulation **(B)** and shedding **(C)** of primary human neutrophils during interaction with *C. parapsilosis*. Flow cytometry gating strategies used to measure the phagocytosis **(D)** and reactive oxygen species **(E)** of primary human neutrophils during interaction with *C. parapsilosis*. Gating strategy was drawn based on the uninfected control neutrophils (except phagocytosis) and mean fluorescence intensity was recorded. The data underlying figure can be found in https://doi.org/10.5281/zenodo.21794379 and the rest in the S1 Data File.
(TIFF)

S4 Fig Multidimensional assessment of host-*C. parapsilosis* interface. Gene ontology enrichment analysis of differentially expressed pathways of macrophages infected with *C. parapsilosis* isolates. Upregulated **(A)** and downregulated **(B)** pathways of macrophages infected with *C. parapsilosis* isolates. Representative confocal images of neutrophils (stained with Hoescht) during interaction with iRFP-expressing *C. albicans* (upper) and without neutrophils (lower) (A). *C. albicans* survival was measured by measuring the MFI of iRFP and scale bar represents 100 µm **(C)**. The mutants carrying Efg1^I280T^ **(D)** and Mnl1^S557T^ **(E)** did not show any growth advantage compared to their pertinent wildtype strains under various in vitro conditions. Biofilm analysis of *C. parapsilosis* mutants lacking transcription factors/kinase involved in biofilm regulation using OD600 **(F)**. Fig G–J show growth rate of wildtype (ATCC 22,019) and the mutants derived from it under various in vitro conditions. Note that all in-vitro and biofilm experiments tested at two-independent colonies of the same mutant for rigor. Since such in vitro experiments showed consistent phenotype among independent colonies, only a single colony was used for comprehensive ex vivo analysis shown in Fig 6. The data underlying this S4A and S4B Fig can be found in https://doi.org/10.5281/zenodo.21799493 and the rest in the S1 Data File.
(TIFF)

S1 Table Complete microbiological data of *C. parapsilosis* isolates collected from outbreaks in Turkey and Brazil denoted by T and B, respectively. (XLSX)

S2 Table Differential expression of *C. parapsilosis* genes in (A) biofilm, and (B) planktonic growth conditions. Differential expression analysis was performed by comparing gene expression BP strains (combining 35B and 1T) relative to LBPs (combining 49B and 29T) at each time point for (A) biofilm and (B) planktonic conditions independently and log2 fold change is denoted here. **(C)** Gene age was estimated using orthologous gene family estimates across 15 species. “Old” genes are those conserved across *Ascomycota* species, “Middle” genes are conserved across CTG and non-CTG *Candida* species and *S. cerevisiae*, “Young” gene are conserved across CTG *Candida* species and “Unique” genes are those unique to *C. parapsilosis.*
(XLSX)

S3 Table Filtered list of all variants predicted to affect protein function, such as missense variants, frameshifts, and changes to start and stop codons. Each strain is marked with a 2 if they are homozygous for the variant, a 1 if they are heterozygous for the variants, and 0 if they are homozygous for the reference. Sheet (ii) lists variants unique to BP strains, i.e., not found in LBP strains. Sheets (iii)–(vi) contain the variants which are unique to LBP strains. Sheet (vii) lists the genes that are only mutated in LBP strains, and sheet (viii) summarizes the type and number of mutations in LBP and BP strains.
(XLSX)

S4 Table Heterozygous variants from 35B that underwent LOH in derived strains, in tabular variant call format (VCF), annotated by SIFT. Heterozygous sites are highlighted in yellow, homozygous reference in green, and homozygous alternate in red.
(XLSX)

S5 Table Gene ontology enrichment analysis of genes containing premature stop codons or frameshift mutations in low biofilm producers compared to high biofilm producers. (XLSX)

S6 Table Gene ontology (GO) analysis of macrophages infected with high and low biofilm producer isolates at 3 h post-infection. (CSV)

S7 Table Gene ontology (GO) analysis of macrophages infected with high and low biofilm producer isolates at 24 h post-infection. (CSV)

S8 Table Gene set enrichment analysis (GSEA) of macrophages infected with BP strains demonstrated “Hallmark IL-6/JAK/STAT3”. (CSV)

S9 Table Gene set enrichment analysis (GSEA) of macrophages infected with BP strains exhibited robust induction of chemokines associated with granulocyte recruitment. (CSV)

S10 Table Macrophages infected with LBP strains displayed a transcriptional profile enriched for genes classically associated with proinflammatory (M1-like) activation at 24 h post-infection. (CSV)

S11 Table Sequence details of oligonucleotides used for manipulation of *C. parapsilosis* isolates using CRISPR-Cas9. (XLSX)

S1 Data Complete dataset used to generate figures in the manuscript. Partial FACS files could be accessed using the following link, https://doi.org/10.5281/zenodo.21794379. Data concerning Figs 1D, 1E, S1D, and S1E are deposited in public depository under the following link https://doi.org/10.5281/zenodo.21799493.
(XLSX)

## Abbreviations

BP: biofilm producer
CFUs: colony-forming units
FLCR: fluconazole-resistant
FLCS: fluconazole-susceptible
GO: Gene Ontology
GSEA: gene set enrichment analysis
iNTS: invasive nontyphoidal *Salmonella*
LBP: low-biofilm-p
LDH: lactate dehydrogenase
MDR: multidrug-resistant
PAMP: pathogen-associated molecular pattern
PCA: principal component analysis
PRRs: pattern recognition receptors
ROS: reactive oxygen species
VCF: variant call format
WGS: whole-genome sequencing
YCP: Y132 carrying *C. parapsilosis*
