# Supplementary material for: Outbreaks of fluconazole-resistant Candida parapsilosis are driven by low-biofilm-producing isolates that emerge under host selection
Source: PLoS Biol. 2026 Sep 1;24(9):e3003973. doi: 10.1371/journal.pbio.3003973 (PMC13557500; doi:10.1371/journal.pbio.3003973)
Supplement: Supplementary file 1 [file pbio.3003973.s017.docx]

July 15, 2026

Würzburg, Germany

Dr. Nonia Pariente
Editor-in-Chief

Dr. Joeph Heitman

Editor

*PLOS Biology*
Ref.: PBIOLOGY-D-26-00894R1

Dear Dr Pariente and Dr. Heitman,

We would like to thank you and the reviewers for your feedback on our manuscript **“Host Adaptation by Reduced Biofilm Formation Drives Outbreak Persistence of *Candida parapsilosis*”.** To address each of the reviewers, we have revised our manuscript by modifying the text and Figures. This includes major revisions to certain sections of our manuscript to improve the clarity and flow of the text and to emphasize our major findings. We have integrated these and other changes to address each point of the reviewers, as detailed in the point-by-point response. For convenience and clarity, all major changes are highlighted using Track Changes in the revised manuscript.

We appreciate the detailed input of each reviewer and the opportunity to revise our manuscript for *PLOS Biology*. Thank you very much for giving us the opportunity to revise our manuscript. Please feel welcome to contact me if you would like any additional information or have any questions. We look forward to hearing from you.

Yours sincerely,

Dr. Amir Arastehfar

Dr. Michael K Mansour

Dr. Clarissa J Nobile

Dr. Wenjie Fang

On behalf of all the authors

**Response to Editor and Reviewers**

**Reviewer #1:**
This manuscript by Daneshnia et al., explores the traits involved in the persistence of fluconazole-resistant Candida parapsilosis isolates associated with global outbreaks. They find that a common trait shared among resistant isolates is a low biofilm producing (LBP) phenotype, which is associated with a transcriptional reprogramming toward "old" conserved genes involved in essential metabolic functions. In contrast to the traditional view of biofilms as promoting persistence and resistance, they find that LBP isolates have enhanced stress tolerance, altered cell wall masking and reduced immune recognition, resulting in altered immune cell interactions. Their findings suggest that there may be a trade-off between biofilm formation and tolerance to in vivo environments. The study is very interesting with broad and important implications. However, inconsistencies throughout make data interpretation difficult, thereby reducing excitement and confidence in the work.

**Response:** We would like to thank reviewer’s kind remarks regarding the implication and importance of the study and meticulous points raised by the reviewer. Below please kindly find responses to your comments.

1. Throughout the manuscript it is unclear how and why the representative isolates were selected. Furthermore, while I understand that it is not possible to sequence every isolate in detail, there is some uncertainty as to whether these selected isolates truly represent the broader population.

**Response:** Thanks for raising this critical concern. Despite we would have been delighted to include a larger number of isolates in our study, as reviewer pointed out we whole-genome sequencing for high number of isolates was cost prohibitive. Therefore, we had to choose those isolates based on biofilm level, susceptibility pattern (fluconazole resistant and susceptible), as well as ERG11 mutation to account for all potential phenotype and genotype differences. We Modified the manuscript and added the logic behind the selection of the isolates in the manuscript to address reviewer’s concern.

2. The overall message is a bit confusing. Figure 3 suggests that LBP is associated with stress tolerance and altered immune masking and Figure 4 suggests LBP is increased with enhanced immune evasion; but then Figure 5 indicates that macrophages that take up LBP isolates are then better able to induce neutrophil killing? The induction of ROS by LBP mutants in Figure 6 is similarly confusing.

**Response:** We truly appreciate your concern, and we should have clarified this point better to prevent confusion. We define immune evasion based on the ability of the macrophages and neutrophils in recognizing and taking up the yeast cells. Under this scenario, immune-evasive isolates are less recognized and less taken up by innate immune cells and indeed as shown in Figure 4, LBPs were less phagocytosed by both macrophages and neutrophils. On the contrary of “extracellular” evasion and while “within” the macrophages, LBPs skew the macrophage immune cell responses toward an M1-like state as judged by our comprehensive RNAseq data. Therefore, immune-evasion refers to the ability of innate immune cells to recognize extracellular yeast cells, whereas Figure 5 refers to dynamic response of macrophages already engulfed the yeast cells (intracellular yeast cells). Relevant changes were added to the text to reflect those definitions.

Regarding the ROS production of LBPs, please note that panels within Figure 6 exclusively deal with mutants, which do not fully mimic the genotype of clinical LBPs, but only mimic the phenotype (lower biofilm). As pointed out in the results section, LBPs carried mutations within numerous genes and deletants included in Figure 6 lack certain genes, therefore, cannot simply mimic exact patterns shown by clinical isolates during encounter with immune cells. Relevant changes were made in the results section to prevent confusion.

3. The data shown in Fig 1E does not adequately represent the text in the figure legend or on lines 177-181. It is recommended that this figure be reworked to emphasize the points the authors want to make. Considering that metabolic rewiring of the LBP isolates is a major emphasis of the manuscript, this is a major weakness.

**Response:** We do appreciate this valuable comment from the reviewer. We do agree and accordingly we have added heatmaps showing the expression of metabolic genes enriched in LBP isolates to reflect the metabolic changes and to overcome this weakness. A new figure was added to Figure 1E to better visualize our transcriptomic observations.

4. There are a significant number of inconsistencies and errors throughout the figures, which make them difficult to interpret at best, and questionable at worst. For example, many plots do not start at zero on the y-axis; several plots that are meant to be comparable are not on the same scale on the y-axis; there is inconsistent coloring, abbreviations, and ordering of variables; the ordering of panels is not sequential; Figure 4E and F appear to be the same panel repeated; Figure 5C requires additional labels.

**Response:** We do understand reviewer’s point and indeed initially we used similar y-axis scale for different panels, but we had to show figure panels in different scales. This was because 1) the differences in values between the figure panels were not on similar scales (for instance linear or log or the quantity of the values reported), and 2) each figure panel shows a completely different immune effector function, whose values are not comparable to previous dataset. For instance, differences in values of phagocytosis, ROS production, and killing of LBPs and BPs by macrophages are drastically distinct. Moreover, while linear scale could appreciably show the difference in macrophage ROS production in linear scale, converting it to log scale would mask this difference. Therefore, we had to use different y-axis scales to better show the differences.

Regarding some figures not starting with zero, we noted that macrophage ROS should be consistent with what presented for neutrophils. We do appreciate the reviewer noting this inconsistency and our new Figure 4B has processed relevant data similar to the rest of immune effector functions. Nonetheless, please note that again to better appreciate the differences, we needed to show this graph on linear scale and the range starts at 80 and ends at 160 (the average of ROS production is around >90) and converting this to log scale obscure the differences between BP and LBP.

Regarding the figure 5C, we added the following for better clarification “Note that the P value is a nominal P-value, while NES is the normalized enrichment score”.

We extensively reviewed the manuscript to check for any errors in abbreviations and coloring and changed wherever mistake was found.

Thanks for noting the error in Figures 4E and 4F. The correct Figure 4F was added.

5. The phrase "normalized to pertinent 'x'" is used throughout the figures. What does this mean?

**Response:** Indeed, this is a very valid point. To clarify pertinent control, we had included a definition of controls in figure legends. Pertinent control refers to control for each experimental condition in each assay performed throughout the manuscript. For instance, we used multiple strains for 3hrs intramacrophage survival experiments and here pertinent control for a given strain refers to CFU of the 1hr intramacrophage of the same isolate.

------------------------------------

Reviewer #2:

The manuscript by Daneshnia et al focuses on charactering fluconazole resistant Candida parapsilosis isolates. The authors show that these isolates have a reduced ability to form biofilms, which leads to increased fitness and altered host-pathogen interactions. While the authors tried to identify the genetics behind this interesting observation, deletions of genes introductions on SNPs etc failed to recapitulate the phenotypes, suggesting that this phenotype is complex and mediated via changes/mutations in multiple pathways. However, there is still novelty, as one would usually assume less biofilm formation would lead to less drug resistance challenging the standard dogma.  Most of the assays focused on resorting biofilm formation, and it would be nice to know whether drug resistance was affected in any of the generated strains. Overall, the manuscript is scientifically robust, well-presented and data analysed appropriately. I have the following comments:

**Response:** Authors are extremely grateful for reviewer’s valuable and critical comments. Below, please kindly find the response to your comments.

*       Do you think this is specific to C. parapsilosis, or do you see the emergence if similar traits in other Candia species. If not, why do you think this has occurred in C. parapsilosis?

**Response:** Thanks for raising this important question. As described in the results and further alluded to in the discussion section, we believe that such LBP phenotype allows *C. parapsilosis* to gain specific features highly advantageous during interaction with innate immune cells and we hypothesized that this might be due to the fact that biofilm formation is a costly process and may restrain dissemination during infection. We have alluded to those hypothesis of biofilm defect-mediated enhanced fitness in the discussion section.

Regarding the generalizability of our findings to other fungal pathogens, we only can hypothesize at this stage and we believe that such phenomenon might also hold true for other fungal pathogens able to cause outbreaks, such as *C. auris*, but this may not hold true for *Candida* pathogens known to endogenously cause infection in humans, i.e. *C. albicans*. Testing this hypothesis requires intensive research and we do hope that results from our study will inspire other mycologists to examine the role of biofilm in outbreak persistence of other fungal pathogens. We briefly expanded this hypothesis to further enrich the discussion section.

*       Compared to other Candida species, how good is C. parapsilosis at forming biofilms as the ODs form the CV assay seem low?

**Response:** Biofilm formation capacity is highly strain-dependent (even in *C. albicans*) and that we have not directly compared the biofilm level of *C. parapsilosis* and *C. albicans*. Nonetheless, previous studies conducted by our colleagues (PMID: 33825680) suggest that *C. parapsilosis* “type strain” biofilm formation level is very comparable to “type strains” of *C. albicans* and *C. dubliniensis*. This information was added to the results section.

*       Do you reintroduce WT ERG11 into the Turkish strains, and if so, what impact did this have on biofilm formation and AMR (i.e. do you need the ERG11 mutation for FLC resistance)?

**Response:** Thanks for raising this extremely valuable comment. To address if Y132F confers biofilm defect, we reverted Erg11^Y132F^ in two low biofilm producer isolates from Turkey (59T and 60T) and simultaneously measured both fluconazole susceptibility and biofilm formation. If wildtype Erg11 confers high biofilm formation, then restoring ERG11 sequence to WT should also restore the biofilm capacity of these two low biofilm producers. As shown in “new” Supplementary Figure 1F, reversion of Y132F reduced fluconazole MIC up to 8-16 folds in both isolates (as expected), while it did not impact the biofilm level at all. These data clearly indicate that fluconazole susceptibility and biofilm formation are decoupled and that other genetic determinants underlie biofilm defect. Therefore, manuscript was revised in accordance with these findings, and a new supplementary figure was added.

*       Do all Cp strains that don't form biofilms exert similar phenotypes (i.e. FLC resistance, increased growth and cell wall modifications)?

**Response:** We thank reviewer for raising this important question. To reach a better understanding regarding the generalizability of low biofilm, we included LBP isolates from both Turkey and Brazil. As microbiological details of all isolates are mentioned in Supplementary Table 1, Turkish LBPs were all fluconazole resistant and carrying Erg11^Y132F^, whilst some of the Brazilian isolates, such as 34B, 56B, and 64B, were all susceptible, WT for ERG11 and “yet were low biofilm formers. Therefore, low biofilm producers could be found across range of susceptibility patterns and found in both fluconazole susceptible and resistant isolates.

Nonetheless, LBP isolates regardless of the country of origin and susceptibility profiles showed similar trends toward cell wall remodeling and stress tolerance/ enhanced growth as shown in Figure 4. Additional clarifications were added to results section to better show the *ERG11* genotype and biofilm level of LBPs.

*       Line 270 were all SNPs per Mb?

**Response:** Thanks indeed for noting this mistake. The SNP data are genome-wide and therefore we deleted Mb.

*       For the phagocytosis assays, when using CFUs this will take into account cell attached to the plastic, given that you show LBPs to express lower levels of adhesins, how did you account for this in your assay?

**Response:** We do appreciate raising such a well-thought criticism. Authors believe that lower adherence to plastic surfaces do not interfere with our macrophage interaction studies for multiple reasons as follows, 1) the entire surface of the wells were covered by macrophages, 2) yeast-macrophages co-cultures were briefly centrifuged so that all yeast cells are in very close contact with macrophages, 3) macrophages were extensively washed with PBS to assure that only intracellular yeast cells were taken into account and to remove non-adherent yeast cells. Absence of any extracellular and nonphagocytosed yeasts were validated by direct inspection of plates under the microscope after each washing step (each well is washed at least 5 times, which has ensured lack of any extracellular yeast cells in all occasions). Therefore, we believe that phagocytosis values were the direct measure of immunoevasion rather than lower adhesion to plastic surfaces. Relevant changes were applied to results section to address this valid concern.

*       Line 483: in the host it is thought that cells form biofilms on mucosa surfaces. Did you do any histology of the organs to look at how the cells were growing in vivo, to see if the in vitro biofilm defect holds in vivo?

**Response:** Truly fascinating questions. Unfortunately, we did not assess the histology sections to investigate how yeast cells grow and if how biofilm capacity changes after designated days post-infection. Nonetheless, authors have already planned a comprehensive study to assess how biofilm level changes in the context of systemic infection passage for which we also planned to extensively study *C. parapsilosis* growth during systemic infection. Upon receiving proper funding, hopefully by the end of this year, we do hope to address such intriguing questions.

*       Likewise, if adhesins are downregulated how do you think the yeast is attaching to mucosa surfaces?

**Response:** This is again another important objective of the same study mentioned above in which we aim to investigate how the parental and in-vivo passage adapted isolates behave toward colonization on proper mucosal surfaces for which we aim to include both ex vivo (CaCo2 and HaCat cell lines, etc.) and gastrointestinal tract colonization models that we have used in our previous studies (PMID: 37772846, PMID: 39545363, and PMID: 41870047).

*       You mention that these phenotypes likely evolve via host adaptation like GI passage in C. albicans. Did you try any lab evolution experiments to see if you could evolve strains with similar phenotypes?

**Response:** As alluded to above, regrettably, the extent of such studies and lack of proper funding and manpower at this stage, prevent us from examining such important questions. Nonetheless, we intend to use extensive systemic infection studies to understand how immune cell pressures impact the biofilm capacity of *C. parapsilosis*, which immunity arm and host cell type drive such drastic phenotypic changes. Additionally, since we use multiple parental strains, application of whole-genome sequencing on in-vivo evolved colonies compared to their original parental strains would allow us to better characterize the main genetic changes driving biofilm modulation, which will be tested in the context of comprehensive functional studies.

*       What did you quantify cell wall staining by microscopy rather that FACS, by FACS you could have quantified 10,000 cells rather than 30 cells?

**Response:** We do agree with reviewer that using flow cytometry would give us the opportunity to more comprehensively interrogate the cell wall architecture of thousands of yeast cells from the same isolate. Due to the comprehensive nature of the study and the workload, we rather cooperated with Dr. Carol Munro, whose laboratory has a longstanding experience with cell wall analysis in diverse pathogenic fungi and uses a well-established and reliable fluorescence microscopy method to characterize the exposure level of major cell wall components of pathogenic *Candida* species.


General comments
*       What does YCP stand for?

**Response:** YCP was defined as FLCR *C. parapsilosis* isolates carrying Y132F in Erg11. Please kindly revisit line 116, where YCP was defined.

*       Some of the figure legends, need slight adjustment as it would seems some data has been moved around in the figure but not in the text description in the legend.

**Response:** We greatly appreciate the reviewer for noting these mistakes. We have thoroughly gone through the figure legends and text to ensure that such mistakes are corrected.

*       I would remove boxes around the letter in the figures. Also, some of the text in the figures is quite hard to read due to the size of the figures.

**Response:** We do appreciate that having high quality images are of paramount importance. While the original images generated for this submission were of high quality, the image qualities dropped upon generating the proof and by direct communication with the submission team, we will ensure that all figures are of high quality prior to publication.

We agree with the reviewer regarding the boxes and therefore removed all the boxes near the figure legends as suggested by the reviewer.

*       What do the dashed lines on Fig 2F indicate?

**Response:** It represents the lowest biofilm formation capacity. Figure legend was modified to reflect this change.

*       Does the ethics cover both human and animal experiments?

**Response:** Thanks for asking this critical and important question. Yes, all animal studies were conducted in IACUC accredited animal facilities and the study protocol received approval (IACUC protocol #2017N000058). This information was added in the methods section.

Regarding human studies, all clinical centers involved in this study have already received ethical approval from local ethic committee members and pertinent information are in methods section. Please kindly read the ethical section at the end of the methods section.

*       FACS plots show red and grenn which is not good for people who are colour blind, consider using other colours. Likewise, some of the shades of green in other figures could be hard to see, so could use other brighter colours.

**Response:** Thanks for raising this critical concern. We completely changed all the FACS plots and made new figures shown in gray colors to make them legible and obvious to all readers. Please find the new FACS plots in revised pertinent Supplementary Figures.


------------------------------------

Reviewer #3:

The manuscript "Host Adaptation by Reduced Biofilm Formation Drives Outbreak Persistence of a Major High Priority Human Fungal Pathogen" by Daneshnia et al. showed an attenuation phenotype of biofilm formation in clinical Candida parapsilosis isolates from diverse genetic backgrounds, which mostly display drug resistance. Furthermore, this low biofilm-producing phenotype allows the fungus to evade the host immune system and persist within the host, although this is not intuitive based on our conventional understanding of biofilm formation and host adaptation. The study is interesting and provides novel perspectives on fungal adaptation against host defense.

The entire manuscript includes a wide range of experiments and analyses to support the authors' findings. While this manuscript is very well written with only very minor concerns, the reviewer has listed the following points and hopes the authors can address them before accepting the manuscript for publication.

**Response:** Authors are extremely humbled by kind appraisal of the reviewer and appreciate valuable suggestions. Below please kindly find the response to your comments.

1. It is understandable that introducing LBP mutations into the BP strains can't phenocopy the LBP phenotypes, as these isolates have evolved within a complex host environment, and biofilm formation is a polygenetic phenotype. Other types of genetic abnormalities should be considered, in addition to mutations, such as gene copy number variations. Are there gene copy number variations across the LBP isolates?

**Response:** This is indeed a great point overlooked by authors and we appreciate the reviewer for raising this important concern. We systematically searched our whole-genome sequencing data and we did not find any certain CNVs shared across the LBPs and BPs and accordingly changed the results (also methods section) as outlined below,

**Results:** Coverage analysis indicates that strain 1T has aneuploidy of chromosomes 4 and 8, having three copies of each rather than the typical two (Supplementary Table 3). Similarly, strain 47T has three copies of chromosome 6 (Supplementary Table 3). Several strains show evidence of previously discovered gene amplifications; including genes such as RTA3 (CPAR2_104610), ARR3 (CPAR2_601050), CDR1B (CPAR2_304370), and ERG11(CPAR2_303740) (Supplementary Table 3). Other genes have been deleted to one or zero copies in several strains (Supplementary Table 3). There are no genes that are amplified or deleted in all LBP strains or in all BP strains (Supplementary Table 3).

**Methods:** Copy number was estimated for all *C. parapsilosis* genes using the short-read alignments. The mean coverage across the gene’s open reading frame was found using BEDTools coverage version 2.29.2 (Quinlan & Hall, 2010). This was normalised by dividing by the modal genome coverage as found using BEDTools genomecov (Quinlan & Hall, 2010) and then multiplying by two to account for ploidy. The data were filtered to keep only genes that had an abnormal copy number, i.e. greater than 2.75x or less than 1.25x, in at least one strain. Chromosome ploidy was used by calculating mean coverage across the entire chromosomes and normalised as above.

2. In this research, the complex mutational makeup containing both mutations associated with drug resistance and host adaptation makes it difficult to delineate the genetic contributions. While C. parapsilosis is generally considered a commensal fungus, have LBP-associated mutations previously been identified in other isolates from healthy individuals (e.g., strain CBS6318 in Pryszcz et al. 2013)?

**Response:** Thanks, and indeed it would be interesting to better understand the implication of biofilm formation capacity among healthy individuals. Unfortunately, we do not have access to those strains but surely will follow this up in our future research to better understand the distribution of LBPs and BPs across *C. parapsilosis* isolates collected from healthy individuals. Understanding this would hopefully help us to infer if LBPs isolated from the healthy donors share the same genotypic make up as those found in our study or if they belong to different genotypes. It would be also important to assess their fitness in the context of in vitro and ex vivo studies along with proper BPs collected from healthy individuals.

3. The reviewer would like to suggest a new title specifying Candida parapsilosis, rather than only "a Major High Priority Human Fungal Pathogen", especially because C. parapsilosis is an emerging fungal pathogen.

**Response:** We thank the reviewer and do agree that the title should be changed as suggested by reviewer. Therefore, we changed the title to reflect this suggestion. The new title reads as “Host Adaptation by Reduced Biofilm Formation Drives Outbreak Persistence of *Candida parapsilosis*”.
