# Supplementary material for: Outbreaks of fluconazole-resistant Candida parapsilosis are driven by low-biofilm-producing isolates that emerge under host selection
Source: PLoS Biol. 2026 Sep 1;24(9):e3003973. doi: 10.1371/journal.pbio.3003973 (PMC13557500; doi:10.1371/journal.pbio.3003973)
Supplement: Supplementary file 2 [file pbio.3003973.s018.docx]

August 7, 2026

Würzburg, Germany

Dr. Nonia Pariente
Editor-in-Chief

Dr. Joeph Heitman

Editor

*PLOS Biology*
Ref.: PBIOLOGY-D-26-00894R1

Dear Dr Pariente and Dr. Heitman,

We would like to thank you and the reviewers for your feedback on our manuscript **“Outbreaks of fluconazole-resistant *Candida parapsilosis* are driven by low-biofilm-producing isolates that emerge under host selection”.** To address each of the reviewers, we have revised our manuscript by modifying the text and Figures. We have integrated these and other changes to address each point of the editors, as detailed in the point-by-point response. For convenience and clarity, all changes are highlighted using Track Changes in the revised manuscript.

We appreciate the detailed input of each reviewer and the opportunity to revise our manuscript for *PLOS Biology*. Thank you very much for giving us the opportunity to revise our manuscript. Please feel welcome to contact me if you would like any additional information or have any questions. We look forward to hearing from you.

Yours sincerely,

Dr. Amir Arastehfar

Dr. Michael K Mansour

Dr. Clarissa J Nobile

Dr. Wenjie Fang

On behalf of all the authors

**Response to Editors**

Thank you for your patience while we considered your revised manuscript "Host Adaptation by Reduced Biofilm Formation Drives Outbreak Persistence of Candida parapsilosis" for publication as a Research Article at PLOS Biology. This revised version of your manuscript has been evaluated by the PLOS Biology editors, and the Academic Editor.

Based on our Academic Editor's assessment of your revision, we are likely to accept this manuscript for publication, provided you satisfactorily address the following data and other policy-related requests.

1) We routinely suggest changes to titles to ensure maximum accessibility for a broad, non-specialist readership, and to ensure they reflect the contents of the paper. Please ensure you change both the manuscript file and the online submission system, as they need to match for final acceptance:

"Outbreaks of fluconazole-resistant Candida parapsilosis are driven by low-biofilm-producing isolates that emerge under host selection"

**Response:** We are thankful for suggesting this new title. The title was changed as kindly suggested.

2) Please add the weblink of the funding agencies in the Financial Disclosure statement in the manuscript details.
**Response:** Please note that we had difficulty with adjusting the funding sources in the system and a submission team member, Narmatha Raju, kindly agreed to update the funding section with weblinks. Please let us know if the issue still persists.

3) Please move the Ethics statement to the first subheading in the Material & Methods section.

**Response:** The ethics statement was moved to the beginning of the Material & Methods section as suggested.

4) Thank you for providing the numerical values for most figures. However, we are missing the data for some figures Figures 1DE, 4G, 5ABC, S1DE, S4ABD-J. In addition, there seems to be something off with the data from the supplemental figures, as there is data for an S6 Figure, but there is no such figure.

**Response:** Indeed, we appreciate the editors for kindly noting the issues with the source data. We corrected this issue.

Regarding the missing data for Figures 4G and 5ABC, please note that they are already listed in the supplementary tables and the source data here only includes the values found in our experiments that are used to generate graphs. This way, the full dataset used in the manuscript is provided. The data for Figures D and E and Figures S1D and S1E were deposited in public depository under the following link https://doi.org/10.5281/zenodo.21799493, whereas Figures S4A is Table S6 and Figure S4B is Table S7.

*You may be aware of the PLOS Data Policy, which requires that all data be made available without restriction: <http://journals.plos.org/plosbiology/s/data-availability>. For more information, please also see this editorial: <http://dx.doi.org/10.1371/journal.pbio.1001797>
NOTE: the numerical data provided should include all replicates AND the way in which the plotted mean and errors were derived (it should not present only the mean/average values).

5) Please cite the location of the data clearly in all relevant main and supplementary Figure legends, e.g. “The data underlying this Figure can be found in S1 Data” or “The data underlying this Figure can be found in <https://doi.org/10.5281/zenodo.XXXXX>”.

**Response:** Information regarding figures without supplementary tables or raw FACS files were deposited in public depository and all figure and table labelling were invariably used throughout the manuscript.

6) Please provide the tree files for the phylogenetic trees in Figures 1F, 2AEF, S2C. Please make sure all relevant figures have scale bars.

**Response:** The tree files with proper scale bars were included during the submission and named as Figure 2A-tree, Figure2E-tree, and Figures 2F and S2 tree. Please note that the citation for this was not included in the manuscript.

7) For figures containing FACS data (Figures 2ABC, 4AB, 6), please provide the FCS files and a picture showing the successive plots and gates that were applied to the FCS files to generate the figure. We ask that you please deposit this data in an open depository like Zenodo and provide the accession number/URL of the deposition in the Data Availability Statement in the online submission form.

**Response:** Thanks for informing us about the requirement for the availability of the FACS files. As per my conversation in an email thread with Dr. Melissa Vázquez Hernández, we unfortunately have a partial record of the FACS files. Nonetheless, those files along with the representative pictures showing their analysis are deposited here, https://doi.org/10.5281/zenodo.21794379. These changes were reflected in the manuscript.

8) Supplementary files (e.g., excel). Please ensure that all data files are uploaded as 'Supporting Information' and are invariably referred to (in the manuscript, figure legends, and the Description field when uploading your files) using the following format verbatim: S1 Data, S2 Data, etc. Multiple panels of a single or even several figures can be included as multiple sheets in one excel file that is saved using exactly the following convention: S1_Data.xlsx (using an underscore).

**Response:** All the figures and tables citations within the text changed as suggested. Please note that for Supplementary Tables, we have two different citations, those with only one sheet were referred to as S1 Table and those with more than 1 sheets as Sx_Table.xlsx (x refers to Table number).

9) Please add a scale bar in the following microscopy pictures in Figure S4

**Response:** Thanks for noting the missing scale bar. The new version of the Figure S4 now has scale bar.

10)  Please ensure that your Data Statement in the submission system accurately describes where your data can be found and is in final format, as it will be published as written there.

**Response:** Certainly.

11) Per journal policy, if you have generated any custom code during the course of this investigation, please make it available without restrictions. Please ensure that the code is sufficiently well documented and reusable, and that your Data Statement in the Editorial Manager submission system accurately describes where your code can be found. More information on our Code Policy, what and how to share can be found here: <https://journals.plos.org/plosbiology/s/code-availability>.

**Response:** Custom code was not generated.
