# Supplementary material for: Outbreaks of fluconazole-resistant Candida parapsilosis are driven by low-biofilm-producing isolates that emerge under host selection
Source: PLoS Biol. 2026 Sep 1;24(9):e3003973. doi: 10.1371/journal.pbio.3003973 (PMC13557500; doi:10.1371/journal.pbio.3003973)
Supplement: Peer review history

# Editor report: Decision Letter 0

**DOI:** 10.1371/journal.pbio.3003973.r001

**Authors:** Melissa Vazquez Hernandez

29 Mar 2026

Dear Dr Arastehfar,

Thank you for submitting your manuscript entitled "Host Adaptation by Reduced Biofilm Formation Drives Outbreak Persistence of a Major High Priority Human Fungal Pathogen" for consideration as a Research Article by PLOS Biology.

Your manuscript has now been evaluated by the PLOS Biology editorial staff as well as by an academic editor with relevant expertise and I am writing to let you know that we would like to send your submission out for external peer review.

However, before we can send your manuscript to reviewers, we need you to complete your submission by providing the metadata that is required for full assessment. To this end, please login to Editorial Manager where you will find the paper in the 'Submissions Needing Revisions' folder on your homepage. Please click 'Revise Submission' from the Action Links and complete all additional questions in the submission questionnaire.

Once your full submission is complete, your paper will undergo a series of checks in preparation for peer review. After your manuscript has passed the checks it will be sent out for review. To provide the metadata for your submission, please Login to Editorial Manager (https://www.editorialmanager.com/pbiology) within two working days, i.e. by Mar 31 2026 11:59PM.

If your manuscript has been previously peer-reviewed at another journal, PLOS Biology is willing to work with those reviews in order to avoid re-starting the process. Submission of the previous reviews is entirely optional and our ability to use them effectively will depend on the willingness of the previous journal to confirm the content of the reports and share the reviewer identities. Please note that we reserve the right to invite additional reviewers if we consider that additional/independent reviewers are needed, although we aim to avoid this as far as possible. In our experience, working with previous reviews does save time.

If you would like us to consider previous reviewer reports, please edit your cover letter to let us know and include the name of the journal where the work was previously considered and the manuscript ID it was given. In addition, please upload a response to the reviews as a 'Prior Peer Review' file type, which should include the reports in full and a point-by-point reply detailing how you have or plan to address the reviewers' concerns.

During the process of completing your manuscript submission, you will be invited to opt-in to posting your pre-review manuscript as a bioRxiv preprint. Visit http://journals.plos.org/plosbiology/s/preprints for full details. If you consent to posting your current manuscript as a preprint, please upload a single Preprint PDF.

Feel free to email us at plosbiology@plos.org if you have any queries relating to your submission.

Kind regards,

Melissa

Melissa Vazquez Hernandez, Ph.D.

Associate Editor

PLOS Biology

mvazquezhernandez@plos.org

# Peer review: Decision Letter 1

**DOI:** 10.1371/journal.pbio.3003973.r002

**Authors:** Melissa Vazquez Hernandez

21 May 2026

Dear Amir,

Thank you for your patience while your manuscript "Host Adaptation by Reduced Biofilm Formation Drives Outbreak Persistence of a Major High Priority Human Fungal Pathogen" was peer-reviewed at PLOS Biology. It has now been evaluated by the PLOS Biology editors, an Academic Editor with relevant expertise, and by three independent reviewers.

In light of the reviews, we would like to invite you to submit a major revision. The reviewers are broadly positive about the novelty and potential importance of the study, but several key issues need to be addressed before the work can be fully assessed. In particular, Reviewer #1 asks that you clarify how representative isolates were selected, strengthen the presentation of the transcriptomic/metabolic-rewiring data, resolve apparent inconsistencies in the immune-evasion model across the figures, and correct the many figure-labeling, scaling, normalization, and presentation issues. Reviewer #2 requests clarification of the relationship between reduced biofilm formation, fluconazole resistance and ERG11, whether similar phenotypes occur in other Candida species or other low-biofilm C. parapsilosis strains, and how the in vitro phenotypes relate to adhesion, phagocytosis, and in vivo growth. Reviewer #3 asks that the genomic analysis consider mechanisms beyond SNPs, including copy-number variation, and that LBP-associated variants be discussed in the context of commensal or reference isolates. We agree that these points are important, and we ask that the revised manuscript address them, including with additional experimental work when needed.

Given the extent of revision needed, we cannot make a decision about publication until we have seen the revised manuscript and your response to the reviewers' comments. Your revised manuscript is likely to be sent for further evaluation by all or a subset of the reviewers.

In addition to these revisions, you will need to complete some formatting changes, which you will receive in a follow up email. A member of our team will be in touch with a set of requests shortly.

We expect to receive your revised manuscript within 3 months. Please email us (plosbiology@plos.org) if you have any questions or concerns, or would like to request an extension.

At this stage, your manuscript remains formally under active consideration at our journal; please notify us by email if you do not intend to submit a revision so that we may withdraw it.

**IMPORTANT - SUBMITTING YOUR REVISION**

Your revisions should address the specific points made by each reviewer. Please submit the following files along with your revised manuscript:

1. A 'Response to Reviewers' file - this should detail your responses to the editorial requests, present a point-by-point response to all of the reviewers' comments, and indicate the changes made to the manuscript.

*NOTE: In your point-by-point response to the reviewers, please provide the full context of each review. Do not selectively quote paragraphs or sentences to reply to. The entire set of reviewer comments should be present in full and each specific point should be responded to individually, point by point.

You should also cite any additional relevant literature that has been published since the original submission and mention any additional citations in your response.

2. In addition to a clean copy of the manuscript, please also upload a 'track-changes' version of your manuscript that specifies the edits made. This should be uploaded as a "Revised Article with Changes Highlighted" file type.

*Re-submission Checklist*

When you are ready to resubmit your revised manuscript, please refer to this re-submission checklist: https://plos.io/Biology_Checklist

To submit a revised version of your manuscript, please go to https://www.editorialmanager.com/pbiology/ and log in as an Author. Click the link labelled 'Submissions Needing Revision' where you will find your submission record.

Please make sure to read the following important policies and guidelines while preparing your revision:

*Published Peer Review*

Please note while forming your response, if your article is accepted, you may have the opportunity to make the peer review history publicly available. The record will include editor decision letters (with reviews) and your responses to reviewer comments. If eligible, we will contact you to opt in or out. Please see here for more details:

https://blogs.plos.org/plos/2019/05/plos-journals-now-open-for-published-peer-review/

*PLOS Data Policy*

Please note that as a condition of publication PLOS' data policy (http://journals.plos.org/plosbiology/s/data-availability) requires that you make available all data used to draw the conclusions arrived at in your manuscript. If you have not already done so, you must include any data used in your manuscript either in appropriate repositories, within the body of the manuscript, or as supporting information (N.B. this includes any numerical values that were used to generate graphs, histograms etc.). For an example see here: http://www.plosbiology.org/article/info%3Adoi%2F10.1371%2Fjournal.pbio.1001908#s5

*Blot and Gel Data Policy*

We require the original, uncropped and minimally adjusted images supporting all blot and gel results reported in an article's figures or Supporting Information files. We will require these files before a manuscript can be accepted so please prepare them now, if you have not already uploaded them. Please carefully read our guidelines for how to prepare and upload this data: https://journals.plos.org/plosbiology/s/figures#loc-blot-and-gel-reporting-requirements

*Protocols deposition*

To enhance the reproducibility of your results, we recommend that if applicable you deposit your laboratory protocols in protocols.io, where a protocol can be assigned its own identifier (DOI) such that it can be cited independently in the future. Additionally, PLOS ONE offers an option for publishing peer-reviewed Lab Protocol articles, which describe protocols hosted on protocols.io. Read more information on sharing protocols at https://plos.org/protocols?utm_medium=editorial-email&utm_source=authorletters&utm_campaign=protocols

Thank you again for your submission to our journal. We hope that our editorial process has been constructive thus far, and we welcome your feedback at any time. Please don't hesitate to contact us if you have any questions or comments.

Sincerely,

Melissa

Melissa Vazquez Hernandez, Ph.D.

Associate Editor

PLOS Biology

mvazquezhernandez@plos.org

------------------------------------

REVIEWERS' COMMENTS

------------------------------------

Reviewer #1:

This manuscript by Daneshnia et al., explores the traits involved in the persistence of fluconazole-resistant Candida parapsilosis isolates associated with global outbreaks. They find that a common trait shared among resistant isolates is a low biofilm producing (LBP) phenotype, which is associated with a transcriptional reprogramming toward "old" conserved genes involved in essential metabolic functions. In contrast to the traditional view of biofilms as promoting persistence and resistance, they find that LBP isolates have enhanced stress tolerance, altered cell wall masking and reduced immune recognition, resulting in altered immune cell interactions. Their findings suggest that there may be a trade-off between biofilm formation and tolerance to in vivo environments. The study is very interesting with broad and important implications. However, inconsistencies throughout make data interpretation difficult, thereby reducing excitement and confidence in the work.

1. Throughout the manuscript it is unclear how and why the representative isolates were selected. Furthermore, while I understand that it is not possible to sequence every isolate in detail, there is some uncertainty as to whether these selected isolates truly represent the broader population.

2. The overall message is a bit confusing. Figure 3 suggests that LBP is associated with stress tolerance and altered immune masking and Figure 4 suggests LBP is increased with enhanced immune evasion; but then Figure 5 indicates that macrophages that take up LBP isolates are then better able to induce neutrophil killing? The induction of ROS by LBP mutants in Figure 6 is similarly confusing.

3. The data shown in Fig 1E does not adequately represent the text in the figure legend or on lines 177-181. It is recommended that this figure be reworked to emphasize the points the authors want to make. Considering that metabolic rewiring of the LBP isolates is a major emphasis of the manuscript, this is a major weakness.

4. There are a significant number of inconsistencies and errors throughout the figures, which make them difficult to interpret at best, and questionable at worst. For example, many plots do not start at zero on the y-axis; several plots that are meant to be comparable are not on the same scale on the y-axis; there is inconsistent coloring, abbreviations, and ordering of variables; the ordering of panels is not sequential; Figure 4E and F appear to be the same panel repeated; Figure 5C requires additional labels.

5. The phrase "normalized to pertinent 'x'" is used throughout the figures. What does this mean?

------------------------------------

Reviewer #2:

The manuscript by Daneshnia et al focuses on charactering fluconazole resistant Candida parapsilosis isolates. The authors show that these isolates have a reduced ability to form biofilms, which leads to increased fitness and altered host-pathogen interactions. While, the authors tried to identify the genetics behind this interesting observation, deletions of genes introductions on SNPs etc failed to recapitulate the phenotypes, suggesting that this phenotype is complex and mediated via changes/mutations in multiple pathways. However, there is still novelty, as one would usually assume less biofilm formation would lead to less drug resistance challenging the standard dogma. Most of the assays focused on resorting biofilm formation, and it would be nice to know whether drug resistance was affected in any of the generated strains. Overall, the manuscript is scientifically robust, well-presented and data analysed appropriately. I have the following comments:

*Do you think this is specific to C. parapsilosis, or do you see the emergence if similar traits in other Candia species. If not, why do you think this has occurred in C. parapsilosis?

*Compared to other Candida species, how good is C. parapsilosis at forming biofilms as the ODs form the CV assay seem low?

*Do you reintroduce WT ERG11 into the Turkish strains, and if so, what impact did this have on biofilm formation and AMR (i.e. do you need the ERG11 mutation for FLC resistance)?

*Do all Cp strains that don't form biofilms exert similar phenotypes (i.e. FLC resistance, increased growth and cell wall modifications)?

*Line 270 were all SNPs per Mb?

*For the phagocytosis assays, when using CFUs this will take into account cell attached to the plastic, given that you show LBPs to express lower levels of adhesins, how did you account for this in your assay?

*Line 483: in the host it si thought that cells form biofilms on mucosa surfaces. Did you do any histology of the organs to look at how the cells were growing in vivo, to see if the in vitro biofilm defect holds in vivo?

*Likewise, if adhesins are downregulated how do you think the yeast is attaching to mucosa surfaces?

*You mention that these phenotypes likely evolve via host adaptation like GI passage in C. albicans. Did you try any lab evolution experiments to see if you could evolve strains with similar phenotypes?

*What did you quantify cell wall staining by microscopy rather that FACS, by FACS you could have quantified 10,000 cells rather than 30 cells?

General comments

*What does YCP stand for?

*Some of the figure legends, need slight adjustment as it would seems some data has been moved around in the figure but not in the text description in the legend.

*I would remove boxes around the letter in the figures. Also, some of the text in the figures is quite hard to read due to the size of the figures.

*What do the dashed lines on Fig 2F indicate?

*Does the ethics cover both human and animal experiments?

*FACS plots show red and grenn which is not good for people who are colour blind, consider using other colours. Likewise, some of the shades of green in other figures could be hard to see, so could use other brighter colours.

------------------------------------

Reviewer #3:

The manuscript "Host Adaptation by Reduced Biofilm Formation Drives Outbreak Persistence of a Major High Priority Human Fungal Pathogen" by Daneshnia et al. showed an attenuation phenotype of biofilm formation in clinical Candida parapsilosis isolates from diverse genetic backgrounds, which mostly display drug resistance. Furthermore, this low biofilm-producing phenotype allows the fungus to evade the host immune system and persist within the host, although this is not intuitive based on our conventional understanding of biofilm formation and host adaptation. The study is interesting and provides novel perspectives on fungal adaptation against host defense.

The entire manuscript includes a wide range of experiments and analyses to support the authors' findings. While this manuscript is very well written with only very minor concerns, the reviewer has listed the following points and hopes the authors can address them before accepting the manuscript for publication.

1. It is understandable that introducing LBP mutations into the BP strains can't phenocopy the LBP phenotypes, as these isolates have evolved within a complex host environment, and biofilm formation is a polygenetic phenotype. Other types of genetic abnormalities should be considered, in addition to mutations, such as gene copy number variations. Are there gene copy number variations across the LBP isolates?

2. In this research, the complex mutational makeup containing both mutations associated with drug resistance and host adaptation makes it difficult to delineate the genetic contributions. While C. parapsilosis is generally considered a commensal fungus, have LBP-associated mutations previously been identified in other isolates from healthy individuals (e.g., strain CBS6318 in Pryszcz et al. 2013)?

3. The reviewer would like to suggest a new title specifying Candida parapsilosis, rather than only "a Major High Priority Human Fungal Pathogen", especially because C. parapsilosis is an emerging fungal pathogen.

# Author comment: Author response to Decision Letter 2

**DOI:** 10.1371/journal.pbio.3003973.r003

22 Jul 2026

Attachment Submitted filename: Detailed response to reviewers comments.docx

# Editor report: Decision Letter 2

**DOI:** 10.1371/journal.pbio.3003973.r004

**Authors:** Melissa Vazquez Hernandez

3 Aug 2026

Dear Amir,

Thank you for your patience while we considered your revised manuscript "Host Adaptation by Reduced Biofilm Formation Drives Outbreak Persistence of Candida parapsilosis" for publication as a Research Article at PLOS Biology. This revised version of your manuscript has been evaluated by the PLOS Biology editors, and the Academic Editor.

Based on our Academic Editor's assessment of your revision, we are likely to accept this manuscript for publication, provided you satisfactorily address the following data and other policy-related requests.

1) We routinely suggest changes to titles to ensure maximum accessibility for a broad, non-specialist readership, and to ensure they reflect the contents of the paper. Please ensure you change both the manuscript file and the online submission system, as they need to match for final acceptance:

"Outbreaks of fluconazole-resistant Candida parapsilosis are driven by low-biofilm-producing isolates that emerge under host selection"

2) Please add the weblink of the funding agencies in the Financial Disclosure statement in the manuscript details.

3) Please move the Ethics statement to the first subheading in the Material & Methods section.

4) Thank you for providing the numerical values for most figures. However, we are missing the data for some figures Figures 1DE, 4G, 5ABC, S1DE, S4ABD-J. In addition, there seems to be something off with the data from the supplemental figures, as there is data for an S6 Figure, but there is no such figure.

*You may be aware of the PLOS Data Policy, which requires that all data be made available without restriction: http://journals.plos.org/plosbiology/s/data-availability. For more information, please also see this editorial: http://dx.doi.org/10.1371/journal.pbio.1001797

NOTE: the numerical data provided should include all replicates AND the way in which the plotted mean and errors were derived (it should not present only the mean/average values).

5) Please cite the location of the data clearly in all relevant main and supplementary Figure legends, e.g. “The data underlying this Figure can be found in S1 Data” or “The data underlying this Figure can be found in https://doi.org/10.5281/zenodo.XXXXX”

6) Please provide the tree files for the phylogenetic trees in Figures 1F, 2AEF, S2C. Please make sure all relevant figures have scale bars.

7) For figures containing FACS data (Figures 2ABC, 4AB, 6), please provide the FCS files and a picture showing the successive plots and gates that were applied to the FCS files to generate the figure. We ask that you please deposit this data in an open depository like Zenodo and provide the accession number/URL of the deposition in the Data Availability Statement in the online submission form.

8) Supplementary files (e.g., excel). Please ensure that all data files are uploaded as 'Supporting Information' and are invariably referred to (in the manuscript, figure legends, and the Description field when uploading your files) using the following format verbatim: S1 Data, S2 Data, etc. Multiple panels of a single or even several figures can be included as multiple sheets in one excel file that is saved using exactly the following convention: S1_Data.xlsx (using an underscore).

9) Please add a scale bar in the following microscopy pictures in Figure S4

10) Please ensure that your Data Statement in the submission system accurately describes where your data can be found and is in final format, as it will be published as written there

11) Per journal policy, if you have generated any custom code during the course of this investigation, please make it available without restrictions. Please ensure that the code is sufficiently well documented and reusable, and that your Data Statement in the Editorial Manager submission system accurately describes where your code can be found. More information on our Code Policy, what and how to share can be found here: https://journals.plos.org/plosbiology/s/code-availability

Please note that we cannot accept sole deposition of code in GitHub, as this could be changed after publication. However, you can archive this version of your publicly available GitHub code to Zenodo. Once you do this, it will generate a DOI number, which you will need to provide in the Data Accessibility Statement (you are welcome to also provide the GitHub access information). See the process for doing this here: https://docs.github.com/en/repositories/archiving-a-github-repository/referencing-and-citing-content

As you address these items, please take this last chance to review your reference list to ensure that it is complete and correct. If you have cited papers that have been retracted, please include the rationale for doing so in the manuscript text, or remove these references and replace them with relevant current references. Any changes to the reference list should be mentioned in the cover letter that accompanies your revised manuscript.

In addition to these revisions, you may need to complete some formatting changes, which you will receive in a follow up email. A member of our team will be in touch with a set of requests shortly. If you do not receive a separate email within a few days, please assume that checks have been completed, and no additional changes are required.

We expect to receive your revised manuscript within two weeks.

To submit your revision, please go to https://www.editorialmanager.com/pbiology/ and log in as an Author. Click the link labelled 'Submissions Needing Revision' to find your submission record. Your revised submission must include the following:

- a cover letter that should detail your responses to any editorial requests, if applicable, and whether changes have been made to the reference list

- a Response to Reviewers file that provides a detailed response to the reviewers' comments (if applicable, if not applicable please do not delete your existing 'Response to Reviewers' file.)

- a track-changes file indicating any changes that you have made to the manuscript.

NOTE: If Supporting Information files are included with your article, note that these are not copyedited and will be published as they are submitted. Please ensure that these files are legible and of high quality (at least 300 dpi) in an easily accessible file format. For this reason, please be aware that any references listed in an SI file will not be indexed. For more information, see our Supporting Information guidelines:

https://journals.plos.org/plosbiology/s/supporting-information

*Published Peer Review History*

Please note that you may have the opportunity to make the peer review history publicly available. The record will include editor decision letters (with reviews) and your responses to reviewer comments. If eligible, we will contact you to opt in or out. Please see here for more details:

https://plos.org/published-peer-review-history/

*Press*

Should you, your institution's press office or the journal office choose to press release your paper, please ensure you have opted out of Early Article Posting on the submission form. We ask that you notify us as soon as possible if you or your institution is planning to press release the article.

*Protocols deposition*

To enhance the reproducibility of your results, we recommend that if applicable you deposit your laboratory protocols in protocols.io, where a protocol can be assigned its own identifier (DOI) such that it can be cited independently in the future. Additionally, PLOS ONE offers an option for publishing peer-reviewed Lab Protocol articles, which describe protocols hosted on protocols.io. Read more information on sharing protocols at https://plos.org/protocols?utm_medium=editorial-email&utm_source=authorletters&utm_campaign=protocols

Please do not hesitate to contact me should you have any questions.

Sincerely,

Melissa

Melissa Vazquez Hernandez, Ph.D.

Senior Editor

mvazquezhernandez@plos.org

PLOS Biology

# Author comment: Author response to Decision Letter 3

**DOI:** 10.1371/journal.pbio.3003973.r005

11 Aug 2026

Attachment Submitted filename: Detailed response to reviewers.docx

# Editor report: Decision Letter 3

**DOI:** 10.1371/journal.pbio.3003973.r006

**Authors:** Melissa Vazquez Hernandez

12 Aug 2026

Dear Amir,

Thank you for the submission of your revised Research Article "Outbreaks of fluconazole-resistant Candida parapsilosis are driven by low-biofilm-producing isolates that emerge under host selection" for publication in PLOS Biology. On behalf of my colleagues and the Academic Editor, Jessica Brown, I am pleased to say that we can in principle accept your manuscript for publication, provided you address any remaining formatting and reporting issues. These will be detailed in an email you should receive within 2-3 business days from our colleagues in the journal operations team; no action is required from you until then. Please note that we will not be able to formally accept your manuscript and schedule it for publication until you have completed any requested changes.

Best of luck for your wife in ther interview.

IMPORTANT: Thank you for the revision. However, I noticed that the location of the data is not cited in each Figure legend, and I could only see it in the legend of Fig S1. Please add the legend "The data underlying this figure can be found in (S1 Data/zenodo link)". I have asked my colleagues to include this request alongside their own.

Please take a minute to log into Editorial Manager at http://www.editorialmanager.com/pbiology/, click the "Update My Information" link at the top of the page, and update your user information to ensure an efficient production process.

PRESS

We frequently collaborate with press offices. If your institution or institutions have a press office, please notify them about your upcoming paper at this point, to enable them to help maximise its impact. If the press office is planning to promote your findings, we would be grateful if they could coordinate with biologypress@plos.org. If you have previously opted in to the early version process, we ask that you notify us immediately of any press plans so that we may opt out on your behalf.

We also ask that you take this opportunity to read our Embargo Policy regarding the discussion, promotion and media coverage of work that is yet to be published by PLOS. As your manuscript is not yet published, it is bound by the conditions of our Embargo Policy. Please be aware that this policy is in place both to ensure that any press coverage of your article is fully substantiated and to provide a direct link between such coverage and the published work. For full details of our Embargo Policy, please visit http://www.plos.org/about/media-inquiries/embargo-policy/.

Thank you again for choosing PLOS Biology for publication and supporting Open Access publishing. We look forward to publishing your study.

Sincerely,

Melissa

Melissa Vazquez Hernandez, Ph.D., Ph.D.

Senior Editor

PLOS Biology

mvazquezhernandez@plos.org
